# Chemodiversity, biosynthetic regulation, and functional roles of *Eucalyptus* phenolics: applications and prospects

**DOI:** 10.3389/fpls.2025.1733676

**Published:** 2026-01-12

**Authors:** Jordán Pérez-Martínez, Carlos Maldonado, Freddy Mora-Poblete

**Affiliations:** 1 Institute of Biological Sciences, University of Talca, Talca, Chile; 2 Centro de Genómica y Bioinformática, Facultad de Ciencias, Universidad Mayor, Huechuraba, Santiago, Chile

**Keywords:** bioavailability, ellagitannins, extraction technologies, metabolic profiling, phenylpropanoid pathway

## Abstract

*Eucalyptus* species constitute a rich reservoir of bioactive phenolic compounds. In contrast to the extensively characterized essential oils, the non-volatile phenolic fraction remains fragmented across the literature and insufficiently integrated from biochemical, ecological, and applied perspectives. This review synthesizes current evidence on the structural diversity, tissue distribution, and biosynthetic regulation of *Eucalyptus* phenolics, and uniquely bridges their ecological functions with their translational relevance in pharmaceutical, agricultural (biopesticides), and environmental applications. Quantitative analyses indicate that lignified organs such as bark and stump wood consistently contain the highest phenolic concentrations, reaching 474.9 mg/g in *E. camaldulensis* bark, whereas foliar tissues show pronounced intra- and interspecific variation driven by extraction polarity. We identify a critical gap in the field: although over 103 phenolic compounds have been documented, many reports lack methodological standardization, quantitative reproducibility, and chemometric alignment, limiting their comparability and downstream industrial translation. Across organs, several dominant phenolics are recurrently identified, including ellagic acid, epicatechin, gallic acid, quercetin-glucuronide, and sinapic acid. Bark of *E. urograndis*, *E. grandis*, and *E. camaldulensis* is especially enriched in ellagitannins and flavan-3-ols, while leaf extracts of *E. globulus* accumulate high levels of glucuronidated flavonols. Distinctive metabolites, including rosmarinic acid, occur only in *E. marginata*. Beyond compositional surveys, this review examines biosynthetic regulation, ecological roles, and emerging technological applications. We highlight that persistent challenges include extraction standardization, metabolic engineering, and the design of biodelivery systems. Overall, this review positions *Eucalyptus* phenolics as strong candidates for innovation, while providing a clearer roadmap to overcome persistent limitations, particularly in compound-specific bioactivities, transcriptional and metabolic regulatory pathways, and genotype–environment–management interactions.

## 1 Introduction

Phenolic compounds are among the most chemically diverse and functionally significant secondary metabolites in plants and are known to affect both ecological adaptation and human health. Across the plant kingdom, they act as key biochemical mediators of stress resilience, protecting against ultraviolet radiation, oxidative damage, herbivory, and pathogen invasion through potent antioxidant and antimicrobial properties (Mahmoud and Elansary, 2024; Goura et al., 2025). Structurally, their hallmark feature, aromatic rings bearing hydroxyl groups, confers a high redox potential that underlies their capacity to scavenge reactive oxygen species (ROS) across biological systems (Yeshi et al., 2022; Rao and Zheng, 2025).

With > 8,000 chemically characterized structures, this class includes phenolic acids, flavonoids, tannins, and lignans, whose functional roles extend across defense, development, and signaling. Many of these molecules also display clinically relevant bioactivities, including anti-inflammatory, anticancer, cardioprotective, and neuroprotective effects, positioning them as promising agents for therapeutic applications and functional food products (Shah et al., 2018; Ali et al., 2023; Mazewski et al., 2019). Given this potential, phenolics have emerged as important phytochemicals, warranting renewed attention for their translational potential not only in human health but also in sustainable agriculture, particularly as biopesticides (Abdelkhalek et al., 2020).

*Eucalyptus* species (Myrtaceae) have high levels of bioactive phenolic compounds, particularly in bark and foliar tissues (Hasni et al., 2021; Lirong et al., 2025). These include flavonoids, phenolic acids such as gallic, ellagic, and caffeic acids, as well as tannins and terpenoid phenolics (Qiujian et al., 2021; Liu et al., 2022). In addition to their roles in abiotic stress tolerance and microbial defense, these metabolites have drawn considerable pharmacological interest. Their accumulation patterns, structural diversity, and wide range of documented bioactivities make *Eucalyptus* an important system for studying ecological adaptation as well as the production of natural product-based therapeutics. The convergence between ecological resilience and therapeutic value is exemplified by phenolic compounds involvement in drought tolerance and pest deterrence in native habitats, coupled with long-standing medicinal use to treat respiratory and inflammatory disorders (Gullón et al., 2020; Sánchez-Loredo et al., 2024). Current research reinforces this therapeutic value, demonstrating that these stress-responsive phytochemicals interact with key metabolic and redox-regulatory pathways in human cells, supported by their strong reactive-oxygen-species-scavenging activity (Álvarez et al., 2021; Moges et al., 2024).

The therapeutic and industrial relevance of *Eucalyptus*-derived phenolics spans multiple sectors, supported by their strong antioxidant and bioactive capacity. In pharmaceuticals, they have been used to manage oxidative stress-related conditions including diabetes, cardiovascular disease, and neurodegenerative disorders (Ali et al., 2023). In food systems, their antimicrobial and radical-scavenging properties support use as natural preservatives with added nutritional benefits (De Oliveira et al., 2025; Liu et al., 2025). Cosmetic industries likewise exploit their antioxidative potential for dermal protection and anti-aging products (Poveda-Giraldo and Cardona Alzate, 2021; Almeida et al., 2019). In the agricultural sector, *Eucalyptus* phenolics are gaining attention as eco-friendly biopesticides, demonstrating significant allelopathic and herbicidal activity (Pinto et al., 2021) and broad-spectrum antibacterial efficacy against crop pathogens (Kahla et al., 2017). Similarly, beyond biomedical applications, materials science has recently utilized these metabolites in UV-protective textiles and environmentally benign corrosion inhibitors, demonstrating their broad cross-disciplinary potential (da Silva et al., 2018; Chaudhry et al., 2024).

Despite such advances, major challenges remain in realizing the biotechnological and therapeutic promise of *Eucalyptus* phenolics. Current priorities include enhancing extraction efficiency while maintaining molecular integrity, improving bioavailability through advanced delivery systems, and adopting sustainable cultivation practices to support scale-up (Álvarez et al., 2021; Rao and Zheng, 2025). This review critically synthesizes current knowledge of diversity, biosynthesis, functional roles, and emerging applications of *Eucalyptus* phenolics, with particular focus on their contribution to plant stress responses and translational relevance to human health. By combining ecological, biochemical, and technological perspectives, this review aims to highlight gaps in knowledge, guide future research, and advance the rational exploitation of these metabolites in health, agriculture, and industry.

## 2 Sources of phenolic compounds in *Eucalyptus* species

The genus *Eucalyptus*, which contains >900 species primarily endemic to Australia, exhibits remarkable ecological plasticity and biochemical diversity (Mengistu et al., 2022; Mieres-Castro et al., 2024). Several species have been introduced and widely cultivated in subtropical, temperate, and semi-arid regions worldwide, valued primarily for their economic utility (e.g., for pulpwood production, furniture manufacturing, ornamental planting, or medicinal use) and rapid growth. Paradoxically, their strong environmental resilience, often considered a secondary trait in cultivation programs, has facilitated their naturalization and, in many regions, their progression to invasive behavior (Ouyang et al., 2022). This global expansion has enabled comparative studies of phenolic accumulation across diverse environments and genetic backgrounds. **Table 1** summarizes the main *Eucalyptus* species assessed for phenolic potential, highlighting native versus introduced ranges, altitude preferences, and climatic conditions, all of which influence phenolic content and yield. These species occur in environments with annual precipitation from 200 to 3,000 mm and climates ranging from hot semi-arid (BSh) and savanna (Aw) to oceanic (Cfb) and subpolar oceanic (Cfc). Such variation implies that selective pressures, drought, UV radiation, or pathogen burden, shape interspecific differences in phenolic allocation. For example, *E. camaldulensis*, adapted to semi-arid zones, may depend on constitutive phenolic synthesis as an oxidative stress defense (Lima et al., 2018). By contrast, species such as *E. obliqua* and *E. saligna*, from mesic habitats, often show lower or more variable foliar phenolic levels (Álvarez et al., 2021; Fischer et al., 2020), suggesting distinct regulatory control or inducibility.

**Table 1:** Native range, rainfall regimes, and Köppen climate classifications of *Eucalyptus* species investigated for their phenolic content and potential bioactive applications.

Eucalyptus species	Common name	Native distribution*	Annual rainfall (mm)	Main climates**	Reference
*E. camaldulensis*	Red gum	QLD, NSW, VIC, SA	200–800	Cfa, Cwa, Cfb, BSh, Aw	(Nezu et al. (2020); Khanal et al. (2025); Shang et al. (2019))
*E. globulus*	Blue gum	TAS, VIC, southern SA, southeastern NSW	600–1500	Cfb	(Admasu et al. (2022); Yimam et al. (2024); López-Sánchez et al. (2021); Boulekbache-Makhlouf et al. (2013); Dezsi et al. (2015))
*E. sideroxylon*	Iron wood, mugga, red-iron bark	NSW, VIC, southern QLD	400–1000	Cfa, Cfb, Csb, Csa	(Okba et al. (2021); Ferguson et al. (2023); Miranda et al. (2016))
*E. nitens*	Snow gum, silver top, shining gum	NSW, VIC, TAS	750–1800	Cfb, Cfa	(Ayala et al. (2019); Rocha-Sepúlveda et al. (2023); Vega et al. (2021); Millaniyage et al. (2022); Cheng et al. (2022))
*E. tereticornis*	Forest red gum, blue gum, red irongum	NSW, QLD, VIC	500–3000	Cfb, Cfa, BSh, Aw, Am, Af	(González et al. (2022); Mao et al. (2024); Khaiper et al. (2025))
*E. grandis*	Rose gum	QLD, NSW, VIC	400–1200	Cfb, Cfa, Cwa, Aw	(Ouyang et al. (2022); Wang et al. (2023); Mao et al. (2024); Li et al. (2024); Dai et al. (2022))
*E. saligna*	Sydney blue gum	NSW, southeastern QLD	700–2000	Cfb, Cfa	(Rojas-Sandoval, 2022); Gauthey et al. (2022); Hambisa et al. (2023))
*E. obliqua*	Messmate	NSW, VIC, TAS	950–2000	Cfc, Cfb, Cfa	(Pritzkow et al. (2020); Weisgerber and Weisgerber (2019); Millaniyage et al. (2022))
*E. cinerea*	Argyle apple, silver dollar Gum, mealy stringybark	NSW, southern VIC	700–1500	Cfb	(Sadraoui Ajmi et al. (2023); Silva et al. (2014); Pauzer et al. (2021))
*E. diversicolor*	Karri	WA	800–1200	Csb	(Zhou et al. (2022); Bhandari et al. (2023); Parsons et al. (2022))

*QLD, Queensland; NSW, New South Wales; VIC, Victoria; SA, South Australia; TAS, Tasmania; WA, Western Australia. **Cfa, humid subtropical, Cwa, humid subtropical with dry winter, Cfb, oceanic, Csb, warm-summer Mediterranean, Csa, hot-summer Mediterranean, Cfc, subpolar oceanic, BSh, hot semi-arid, Aw, tropical savanna, Am, tropical monsoon.

### 2.1 Analytical strategies for phenolic profiling in *Eucalyptus*

The accurate assessment of *Eucalyptus* chemodiversity relies heavily on the resolution and sensitivity of the analytical instrumentation employed. While spectrophotometric assays (e.g., Folin-Ciocalteu) provide initial estimates of total phenolic content, they offer no compound-level discrimination and are unsuitable for qualitative or quantitative metabolomic resolution, limiting their interpretability relative to targeted or untargeted MS-based workflows (Chaudhry et al., 2024; Hasni et al., 2021). Establishing detailed inventories of phenolic constituents requires chromatographic separation coupled with selective and high-resolution detection systems. High-Performance Liquid Chromatography (HPLC) coupled with Diode Array Detection (DAD) or Variable Wavelength Detection (VWD) remains the standard for quantifying well-characterized phenolic acids, flavonoids, and simple tannin derivatives, although its resolving power can be limited for some structurally similar or co-eluting compounds. For instance, Abdelkhalek et al. (2020) utilized HPLC-VWD at 284 nm to quantify major markers like gallic acid and rutin in *E. camaldulensis* bark, while Chaudhry et al. (2024) employed RP-HPLC-DAD to profile free and bound phenolics in *E. tereticornis* aerial parts. However, UV-based detection is inherently constrained by its reliance on commercially available standards and by the frequent co-elution or spectral overlap of structurally similar compounds, which limits unambiguous identification. To overcome these limitations and elucidate complex structures such as ellagitannins and glycosylated flavonoids, Mass Spectrometry (MS) is fundamental. Liquid Chromatography coupled to Electrospray Ionization Mass Spectrometry (LC-ESI-MS) allows for putative or level-2 identification based on mass-to-charge (*m/z*) ratios and fragmentation patterns, as demonstrated in the profiling of *E. marginata* leaves (Djebbi et al., 2024; Hasni et al., 2021). More recently, advanced platforms like HPLC-DAD-QTOF-MS, integrating diode-array spectral data with high-resolution accurate-mass detection, have been applied to *E. globulus* to identify putatively blood–brain-barrier-permeable phenolics with high mass accuracy (Romano et al., 2025). Furthermore, technological advancements such as Ultra-High Performance Liquid Chromatography (UHPLC) coupled to tandem MS (MS^n^) have drastically reduced analysis times while increasing chromatographic resolution and peak capacity, particularly for chemically crowded matrices such as *Eucalyptus* wood extracts (Santos et al., 2012). The choice of extraction method, ranging from conventional maceration to ultrasound-assisted extraction (UAE) and pressurized liquid extraction (PLE), also critically influences the phytochemical profile obtained, reinforcing the need for standardized reporting of solvent composition, extraction kinetics, and operating conditions (Domínguez-Rodríguez et al., 2024; Hasni et al., 2021; Lirong et al., 2025).

### 2.2 Phenolic content by tissue and species

As shown in **Table 2**, a clear trend emerges, with lignified tissues such as bark and stump wood consistently showing the highest total phenolic content (TPC), surpassing that of leaves and reproductive organs. Stump wood of *E. globulus* reached 460 ± 5.61 mg/g under ethanolic extraction, with similar yields from methanolic and hydroalcoholic systems (Luís et al., 2014). Bark of *E. camaldulensis* accumulated 474.9 ± 34 mg/g in a 50:50 ethanol–water mixture (Lima et al., 2018), while *E. sideroxylon* bark yielded 440.7 ± 35.15 mg/g with 50% ethanol (Miranda et al., 2016). These concentrations surpass the highest foliar TPCs by more than threefold, underscoring the metabolic richness of periderm tissues, which function in protection, environmental buffering, and secondary metabolite deposition. The limited use of woody biomass in phytochemical valorization is thus striking, given the abundance of bark as a forestry by-product and its relatively low extraction cost. The repeated observation of high bark TPC across species such as *E. globulus*, *E. camaldulensis*, and *E. sideroxylon* suggests convergent metabolic investment, likely shaped by common ecological pressures including UV exposure, water limitation, and herbivory. These findings support prioritizing industrial recovery of bark phenolics, especially from plantation residues.

**Table 2:** Total phenolic content (TPC) reported for different *Eucalyptus* species.

Eucalyptus species	TPC (mg/g dry weight)	Solvent	Plant tissue	Reference
*E. camaldulensis*	46.56 ± 1.05	Acetone (70%)	Leaves	(Nasr et al. (2019))
*E. camaldulensis*	42.47 ± 1.18	Methanol (70%)	Buds	(Nasr et al. (2019))
*E. camaldulensis*	36.66 ± 0.07	Acetone (70%)	Capsules	(Nasr et al. (2019))
*E. camaldulensis*	41.60 ± 1.1	Acetone (30%)	Seeds	(Nasr et al. (2019))
*E. camaldulensis*	49.73 ± 4.73	Methanol (99–100%)	Leaves	(Álvarez et al. (2021))
*E. camaldulensis*	474.9 ± 34	Ethanol/water (50/50%)	Bark	(Lima et al. (2018))
*E. camaldulensis*	148. 6 ± 3.15	Methanol	Leaves	(Ashraf et al. (2015))
*E. camaldulensis*	75.75 ± 2.87	Chloroform	Leaves	(Ashraf et al. (2015))
*E. camaldulensis*	39.13 ± 0.3	Hexane	Leaves	(Ashraf et al. (2015))
*E. camaldulensis*	9.68 ± 3.9	Ethanol (70%)	Leaves	(Wong Paz et al. (2015))
*E. tereticornis*	39.03 ± 0.14	Methanol (80%)	Stem	(Chaudhry et al. (2024))
*E. tereticornis*	80.56 ± 0.88	Methanol (80%)	Seed	(Chaudhry et al. (2024))
*E. leucoxylon*	32.8 ± 0.34	Methanol (80%)	Leaves	(Chograni et al. (2020))
*E. globulus*	27.2 ± 0.9	Methanol (80%)	Leaves	(Chograni et al. (2020))
*E. globulus*	26.88 ± 2.14	Methanol (99–100%)	Leaves	(Álvarez et al. (2021))
*E. globulus*	12.98 ± 0.01	Methanol (70%)	Leaves	(Dos Santos Ferreira et al. (2016))
*E. globulus*	92.9 ± 1	Ethanol (56%)	Leaves	(Gullón et al. (2017))
*E. globulus*	36.9 ± 1.26	Methanol (51%)	Bark	(González et al. (2017))
*E. globulus*	79.4 ± 2	Ethanol (56%)	Leaves	(Gullón et al. (2019))
*E. globulus*	88.34	Methanol (100%)	Leaves	(Santos et al. (2011))
*E. globulus*	156.5 ± 10.4	Ethanol (90%)	Leaves	(Park et al. (2023))
*E globulus*	169.3 ± 12.2	Ethanol (100%)	Leaves	(Park et al. (2023))
*E. globulus*	382.5 ± 4.1	Ethanol (75%)	Stump bark	(Luís et al. (2014))
*E. globulus*	444.6 ± 2.55	Ethanol (75%)	Stump wood	(Luís et al. (2014))
*E. globulus*	451.1 ± 4.1	Methanol	Stump wood	(Luís et al. (2014))
*E. globulus*	460 ± 5.61	Ethanol	Stump wood	(Luís et al. (2014))
*E. globulus*	131.59	Acetone (70%)	Leaves	(Boulekbache-Makhlouf et al. (2013))
*E. urograndis*	56.92 ± 1.18	Methanol (50%)	Bark	(Santos et al. (2012))
*E. maidenii*	26.97 ± 0.58	Methanol (50%)	Bark	(Santos et al. (2012))
*E. sideroxylon*	440.7 ± 35.15	Ethanol (50%)	Bark	(Miranda et al. (2016))
*E. sideroxylon*	38.5 ± 1.4	Methanol (80%)	Leaves	(Chograni et al. (2020))
*E. saligna*	186.1 ± 1.43	Pressurized ethanol (95%)	Leaves	(Chaudhry et al. (2024))
*E. obliqua*	20.82 ± 0.68	Ethanol (99–100%)	Leaves	(Álvarez et al. (2021))
*E. nitens*	46.7 ± 2.01	Methanol (51%)	Bark	(González et al. (2017))
*E. nitens*	112.57 ± 1.75	Ethanol (99–100%)	Leaves	(Álvarez et al. (2021))
*E. nitens*	124.17 ± 6.46	Methanol (32–42%)	Leaves	(Álvarez et al. (2021))
*E. nitens*	36.21 ± 7.15	Water	Leaves	(Álvarez et al. (2021))
*E. cinerea*	59.25 ± 0.38	EtOH-H2O (80%)	Leaves	(Kahla et al. (2017))
*E. cinerea*	70.09 ± 0.08	EtOAc-F	Leaves	(Kahla et al. (2017))
*E. cinerea*	62.07 ± 0.48	ButOH-F	Leaves	(Kahla et al. (2017))
*E. diversicolor*	31.8 ± 1.1	Methanol (80%)	Leaves	(Chograni et al. (2020))

Data were compiled from peer-reviewed studies published between 2011 and 2024.

By contrast, leaf tissues show broader variability, shaped by both tissue plasticity and the choice of solvent system. In *E. globulus*, leaf TPC ranged from 12.98 ± 0.01 mg/g (70% methanol; Dos Santos Ferreira et al., 2016) to 169.3 ± 12.2 mg/g (100% ethanol; Park et al., 2023), with intermediate yields such as 131.59 mg/g using 70% acetone (Boulekbache-Makhlouf et al., 2013). This nearly 13-fold range within a single species illustrates the combined effects of solvent polarity and extraction parameters. Likewise, *E. camaldulensis* leaf extracts varied from 9.68 to 148.6 mg/g depending on solvent, spanning polar alcohols (methanol, ethanol) to less polar systems like chloroform and hexane (Wong Paz et al., 2015; Ashraf et al., 2015).

These solvent-dependent disparities confirm that midpolarity aqueous mixtures (typically 50%–80% ethanol or methanol) generally extract phenolics more efficiently than either pure solvents or low-polarity organics. This pattern holds across species and tissues. For instance, in *E. nitens*, ethanol (99%–100%) produced 112.57 ± 1.75 mg/g from leaves, while water extraction yielded only 36.21 ± 7.15 mg/g (Álvarez et al., 2021). Thus, optimizing extraction protocols is a decisive factor for both biological interpretation and commercial application.

The phenolic potential of nonfoliar, nonlignified tissues is also often overlooked. In *E. camaldulensis*, seeds (41.60 ± 1.1 mg/g), buds (42.47 ± 1.18 mg/g), and capsules (36.66 ± 0.07 mg/g) contain appreciable TPC (Nasr et al., 2019), while *E. tereticornis* seeds reached 80.56 ± 0.88 mg/g (Chaudhry et al., 2024). Although lower than bark, these values reveal reproductive tissues as steady contributors to the phenolic pool, possibly linked to protective functions in seed dispersal and early development. Their compositional profiles warrant closer study, particularly for rare or tissue-specific phenolics with pharmacological promise.

Ecological patterns add further context. Species native to xeric or variable environments, such as *E. camaldulensis* and *E. sideroxylon*, consistently display elevated bark and leaf phenolic levels, probably as constitutive adaptations to oxidative and osmotic stress. In contrast, mesic species like *E. obliqua* and *E. maidenii* show much lower TPC values under comparable conditions (20.82 ± 0.68 and 26.97 ± 0.58 mg/g, respectively), implying reduced basal allocation to phenolic defense (Álvarez et al., 2021; Santos et al., 2012). These patterns align with broader ecological phytochemistry, where abiotic stress gradients drive differential investment in phenolic metabolism.

Overall, the data shown in **Table 2** demonstrate that phenolic yield in *Eucalyptus* is shaped by the interplay of species identity, organ specialization, ecological origin, and extraction method. Moreover, including less-studied taxa such as *E. leucoxylon* (32.8 ± 0.34 mg/g) (Chograni et al., 2020), *E. diversicolor* (31.8 ± 1.1 mg/g) (Chograni et al., 2020), and *E. cinerea* (59.25–70.09 mg/g) (Kahla et al., 2017) provides useful baselines for future comparisons. Expanding chemical profiling in these species, combined with rigorous standardization of extraction conditions, will be essential for realizing the full value of *Eucalyptus*-derived phenolics in pharmaceutical, nutraceutical, and biotechnological domains. It is worth noting that while the volatile composition (essential oils) has been extensively characterized across a vast number of *Eucalyptus* species (Salehi et al., 2019), comprehensive and quantitatively comparable profiling of non-volatile phenolic fraction remains largely confined to a limited subset of species. This distinction underscores a persistent knowledge gap in the chemical characterization of the genus, particularly regarding its structurally diverse hydrolyzable tannins, flavonoids, and phenolic acids.

## 3 Constituent profiles and distribution patterns of *Eucalyptus* phenolic compounds

**Figure 1:**
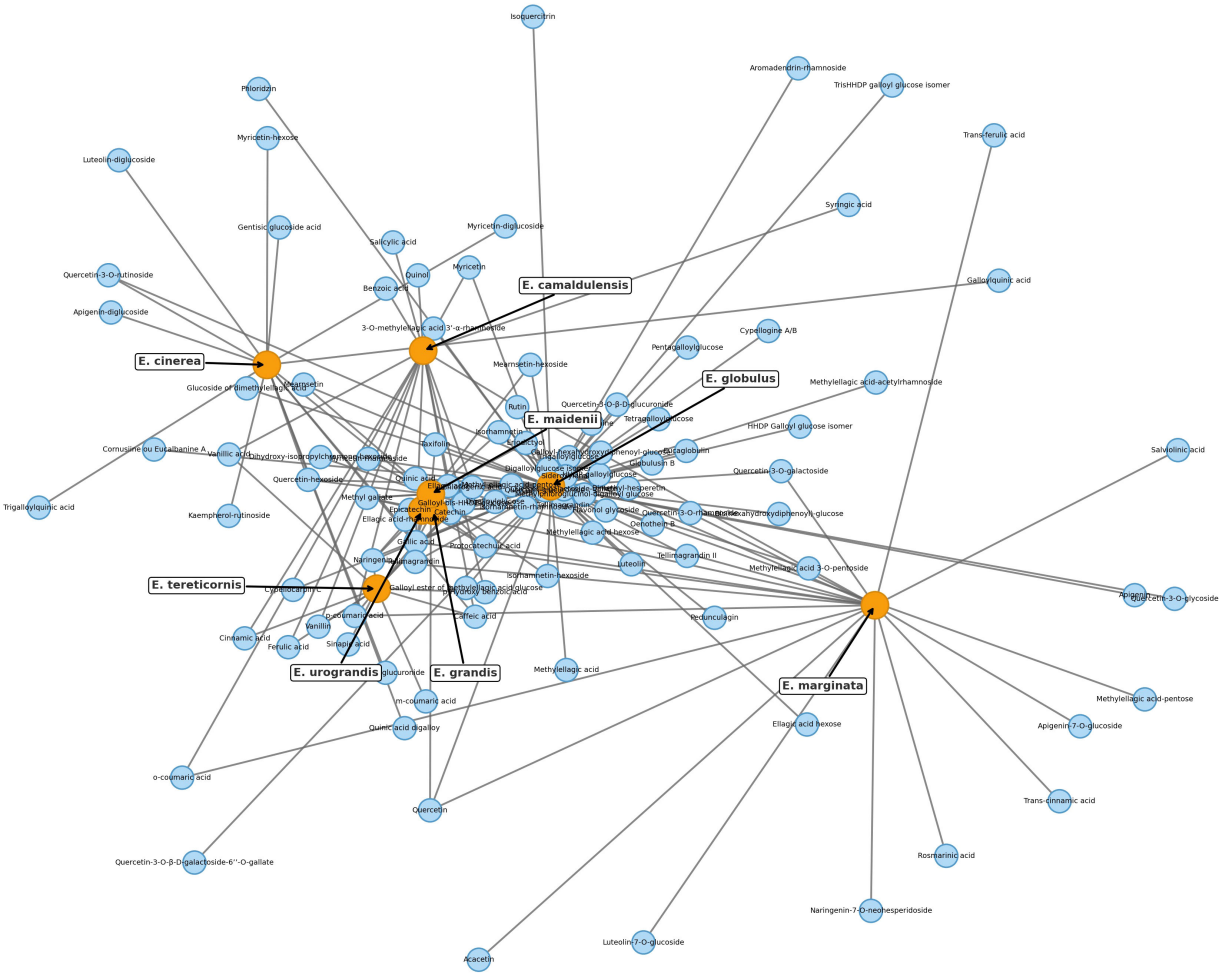
Bipartite cooccurrence networks of phenolic compounds identified in *Eucalyptus* species. Each network connects phenolic compounds (blue nodes) with the species (orange nodes) in which they occur, based on curated reports from leaves, bark, testa, seeds, and stems (see **Supplementary File 1** for the full dataset; primary data sources include Santos et al., 2011, 2012; Boulekbache-Makhlouf et al., 2013; and Chaudhry et al., 2024). Node size reflects the number of reported compounds (degree centrality), underscoring *E. globulus* and *E. camaldulensis* as the most extensively characterized species to date. Node placement was optimized with a force-directed spring layout to enhance clarity. Networks were constructed in Python using NetworkX (v2.8), defining species and compounds as distinct node classes. These diagrams reveal both broadly shared and species-specific phenolic profiles within the genus.

Phenolic composition in *Eucalyptus* reflects a complex interplay of species-specific biosynthetic capacity, tissue specialization, and methodological differences in compound detection. While TPC provides a broad index of phytochemical richness, compound-level profiles offer essential insights into functional, ecological, and chemotaxonomic dimensions of the genus. Here, we integrate compound-specific quantifications (**Supplementary File 1**) with the species–compound co-occurrence network (**Figure 1**) to highlight metabolic features that are both shared and unique across lineages and organs.

In addition to species-specific and tissue-dependent factors, the developmental stage of the tree significantly modulates phenolic composition. A recent metabolomic study on *E. globulus* revealed that leaf extracts from young trees contained considerably higher levels of hydrolyzable tannins, particularly gallic and ellagic acid derivatives, compared to mature trees (Pinto et al., 2022). This age-dependent accumulation likely reflects an adaptive strategy, where juvenile plants allocate more resources to phenolic-based chemical defenses against herbivory, whereas mature trees shift metabolic investment toward other secondary metabolite classes, such as monoterpenes and structural phenolics.

### 3.1 Network analysis and chemotaxonomic markers

The bipartite network depicts a structured chemical landscape among eight *Eucalyptus* species, reflecting both phylogenetic relationships and metabolic convergence. Four species, *E. globulus*, *E. maidenii*, *E. grandis*, and *E. urograndis*, emerge as central hubs, each sharing numerous phenolic compounds with multiple taxa. Their extensive metabolite repertoires, which include ellagitannins, flavonol glycosides, and hydroxycinnamic acids across bark and leaf tissues, suggest conserved biosynthetic capacities coupled with broad ecological plasticity. Notably, *E. grandis*, *E. urograndis*, and *E. maidenii* form a tightly connected cluster enriched in phenolic glycosides such as ellagic acid-rhamnoside, isorhamnetin-rhamnoside, and 3-O-methylquercetin, suggesting a shared ancestry and conserved chemical traits. The strong linkage between *E. grandis* and *E. urograndis* is consistent with their hybrid origin and overlapping phenolic repertoires. In contrast, *E. marginata* stands chemically isolated, producing a suite of unique compounds, including rosmarinic acid, apigenin-7-O-glucoside, and naringenin-7-O-neohesperidoside, restricted to its leaf extracts (Hasni et al., 2021; Djebbi et al., 2024). This metabolic singularity likely reflects phylogenetic divergence and ecological specialization in the southwestern Australian flora. *E. camaldulensis* and *E. tereticornis*, though less central, show specific associations with syringic acid, ferulic acid, and phloroglucinol derivatives, consistent with their adaptation to arid environments and possible lineage-specific defense strategies. Overall, the network supports a dual architecture: a chemically connected core of generalist species with extensive metabolite sharing, and peripheral specialists with narrow, distinctive profiles. These patterns provide insight into the evolution of phenolic metabolism and inform species selection for bioactive discovery.

This analysis further confirms *E. globulus* as the most chemically connected species, linked to >65 phenolic compounds in bark and leaf tissues. These include canonical flavonoids such as quercetin, catechin, and apigenin, alongside hydroxycinnamic acids and phloroglucinol derivatives like globulusin B and eucaglobulin, the latter considered chemotaxonomic markers of section *Globulares* (Boulekbache-Makhlouf et al., 2013; Dos Santos Ferreira et al., 2016). Conversely, *E. marginata* maintains a distinctive fingerprint, with at least nine metabolites reported solely in its leaves, including rosmarinic acid, apigenin-7-O-glucoside, and naringenin-7-O-neohesperidoside, none found in other profiled taxa (Djebbi et al., 2024; Hasni et al., 2021). This distinctive suite underscores species-specific biosynthetic specialization, likely shaped by evolutionary and ecological pressures on phenolic metabolism.

### 3.2 Profiles of major phenolic classes

Hydroxybenzoic and hydroxycinnamic acids, including gallic, ellagic, chlorogenic, ferulic, syringic, and quinic acids, are among the most frequently reported compounds across *Eucalyptus* species and tissues (**Supplementary File 1**; **Table 3**). Their broad occurrence suggests conserved functions in redox homeostasis and defense signaling. Notably, gallic acid reaches its highest concentration in *E. globulus* leaves (142 ± 9 mg/g; Romano et al., 2025), far exceeding levels in *E. tereticornis* testa (23.47 ± 1.25 mg/g; Chaudhry et al., 2024 and bark of *E. urograndis*, *E. grandis*, and *E. maidenii* (Santos et al., 2012). Chlorogenic acid, by contrast, is enriched in *E. cinerea* and *E. maidenii* leaves, with additional accumulation in bark, consistent with its dual antioxidant and antimicrobial activities (Djebbi et al., 2024; Grichi et al., 2025). These phenolic acids also serve as precursors for ellagitannins and hydrolyzable tannins, functioning as both metabolic intermediates and active defense molecules (Zhang et al., 2022; Kaur and Kaur, 2022).

**Table 3:** Main phenolic compounds identified in different organs of *Eucalyptus* species.

Eucalyptus species	Tissue	Main compound	Analytical method	Reference
*E. camaldulensis*	Bark	Rutin 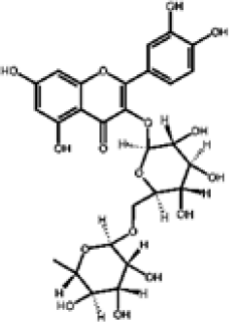 Ellagic acid 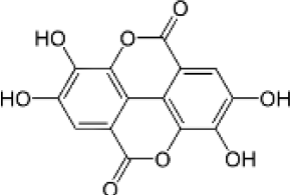	HPLC-VWD (UV 284 nm)	(Abdelkhalek et al. (2020))
*E. cinerea*	Bark	Apigenin-diglucoside 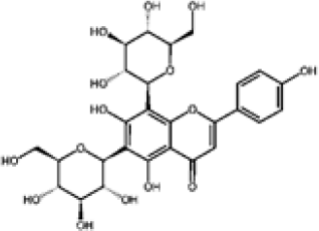 Quercetin-3-O-rutinoside (rutin) 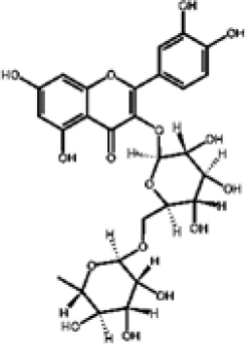	LC-ESI-MS	(Grichi et al. (2025))
*E. globulus*	Bark	Digalloylglucose 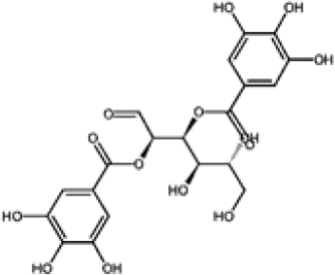 Galloyl-HHDP-glucose 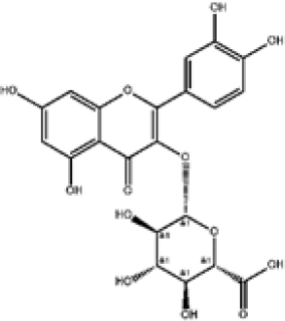	HPLC-DAD-ESI-MS	(Santos et al. (2011))
*E. globulus*	Leaves	Quercetin-glucuronide 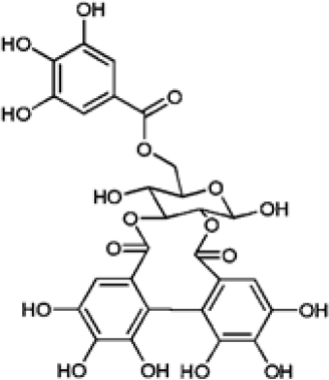 Ellagic acid 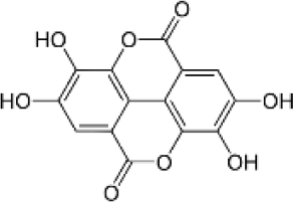	HPLC-DAD-QTOF-MS HPLC-DAD-ESI-MS	(Romano et al. (2025); Boulekbache-Makhlouf et al. (2013))
*E. grandis*	Bark	Ellagic acid-rhamnoside 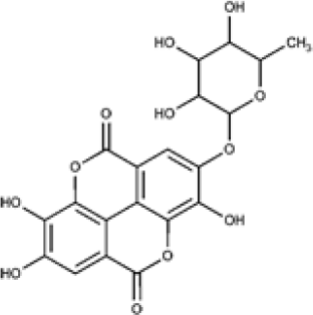 Epicatechin 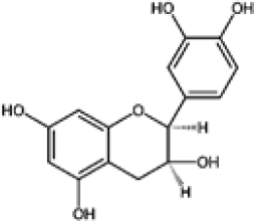 Catechin	HPLC-DAD-ESI-MS/MS	(Santos et al. (2012))
*E. maidenii*	Bark	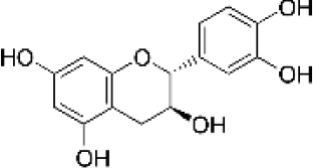 Chlorogenic acid 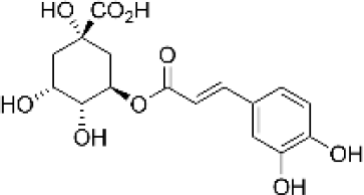	HPLC-DAD-ESI-MS/MS	(Santos et al. (2012))
*E. marginata*	Leaves	Trans-cinnamic acid 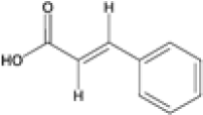 Quercetin-3-O-rhamnoside 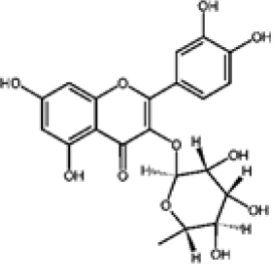	LC-ESI-MS LC-ESI-MS	(Djebbi et al. (2024); Hasni et al. (2021))
*E. tereticornis*	Leaves	Caffeic acid 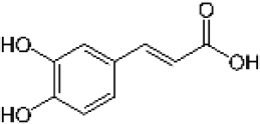 Ferulic acid 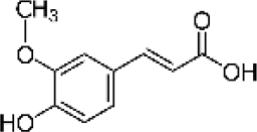	RP-HPLC-DAD	(Chaudhry et al. (2024))
*E. tereticornis*	Seeds	p-Coumaric acid 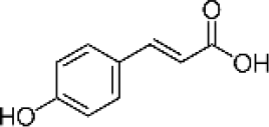 Ferulic acid 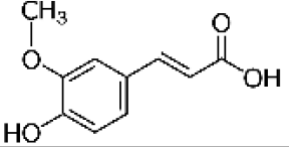	RP-HPLC-DAD	(Chaudhry et al. (2024))
*E. tereticornis*	Stem	Sinapic acid 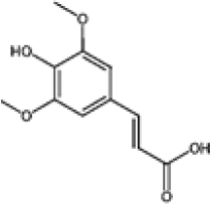 p-Hydroxy benzoic acid 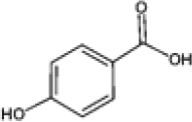	RP-HPLC-DAD	(Chaudhry et al. (2024))
*E. tereticornis*	Testa	Gallic acid 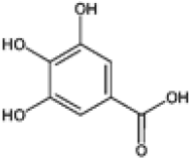 Sinapic acid 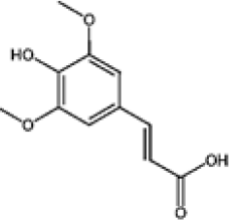	RP-HPLC-DAD	(Chaudhry et al. (2024))
*E. urograndis*	Bark	Galloyl-bis-HHDP-glucose 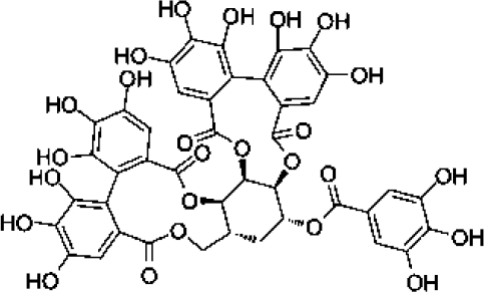 Epicatechin 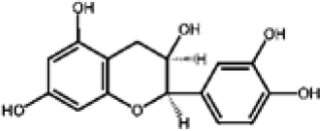	HPLC-DAD-ESI-MS/MS	(Santos et al. (2012))

Flavonoids represent the most chemically diverse and ecologically informative phenolic class in *Eucalyptus*, with species- and tissue-specific profiles shaped by biosynthetic specialization and environmental influence. Epicatechin, a flavan-3-ol with strong antioxidant activity, is consistently detected in bark of *E. urograndis* and *E. grandis*, reaching 118.86 ± 0.71 mg/g and 68.2 ± 0.67 mg/g, respectively (Santos et al., 2012). These findings highlight a conserved role for epicatechin in woody tissues associated with structural defense. In leaves, *E. globulus* stands out as chemically rich, uniquely accumulating quercetin-glucuronide (Romano et al., 2025), a conjugated flavonol with enhanced bioavailability and anti-inflammatory potential. Despite close taxonomic relationships, *E. urograndis* and *E. globulus* display distinct flavonoid glycoside signatures. *E. globulus* leaves are enriched in quercetin-glucuronide and isorhamnetin-rhamnoside, showing a tendency toward glucuronidation and rhamnosylation of flavonols (Romano et al., 2025; Santos et al., 2012). In contrast, *E. urograndis*, along with *E. grandis* and *E. maidenii*, accumulates ellagic acid-rhamnoside in bark, while isorhamnetin-rhamnoside is predominant in bark of *E. urograndis* and *E. grandis* (Santos et al., 2012). Notably, glucuronidated flavonoids are absent in *E. urograndis*, underscoring metabolic divergence even among closely related taxa. This divergence reflects lineage- and organ-specific glycosylation patterns, cautioning against extrapolation of metabolite profiles and reinforcing the need for direct metabolomic characterization to capture species- and tissue-specific phenolic architectures.

Taxifolin (dihydroquercetin) is a flavanonol with a dihydroxyphenyl moiety and a saturated C-ring that confers higher antioxidant activity relative to other flavonoids. Its unique structure enables efficient radical scavenging and redox modulation. Originally isolated from Douglas fir bark, it also occurs in foods such as onions and olive oil, and has attracted attention for its antioxidant, anti-inflammatory, and antimicrobial properties with broad pharmacological value (Liu et al., 2023). However, limited bioavailability remains a major barrier to therapeutic use (Das et al., 2021). Within *Eucalyptus*, taxifolin is reported in bark of *E. globulus* (1.48 mg/g) and *E. maidenii* (4.91 mg/g), suggesting a localized protective role in lignified tissues exposed to stress, desiccation, or microbial challenge (Santos et al., 2011, 2012). These observations underscore the importance of targeted metabolite profiling to uncover ecologically and biotechnologically relevant compounds within the genus.

Other organ-specific profiles are evident. Ellagic acid-rhamnoside occurs exclusively in bark of *E. grandis* (47.32 ± 0.4 mg/g), *E. urograndis* (10.81 ± 0.15 mg/g), and *E. maidenii* (3.13 ± 0.13 mg/g) (Santos et al., 2012). Similarly, eriodictyol, a flavanone with potent antioxidant and anti-inflammatory activities, is restricted to bark of *E. maidenii* (0.93 ± 0.04 mg/g) and *E. globulus* (6.9 mg/g) (Santos et al., 2011). These bark-specific metabolites play critical roles in protecting lignified tissues from oxidative stress and microbial invasion, particularly under conditions of injury or environmental fluctuation (Fontaine et al., 2017; Yin et al., 2024).

**Figure 2:**
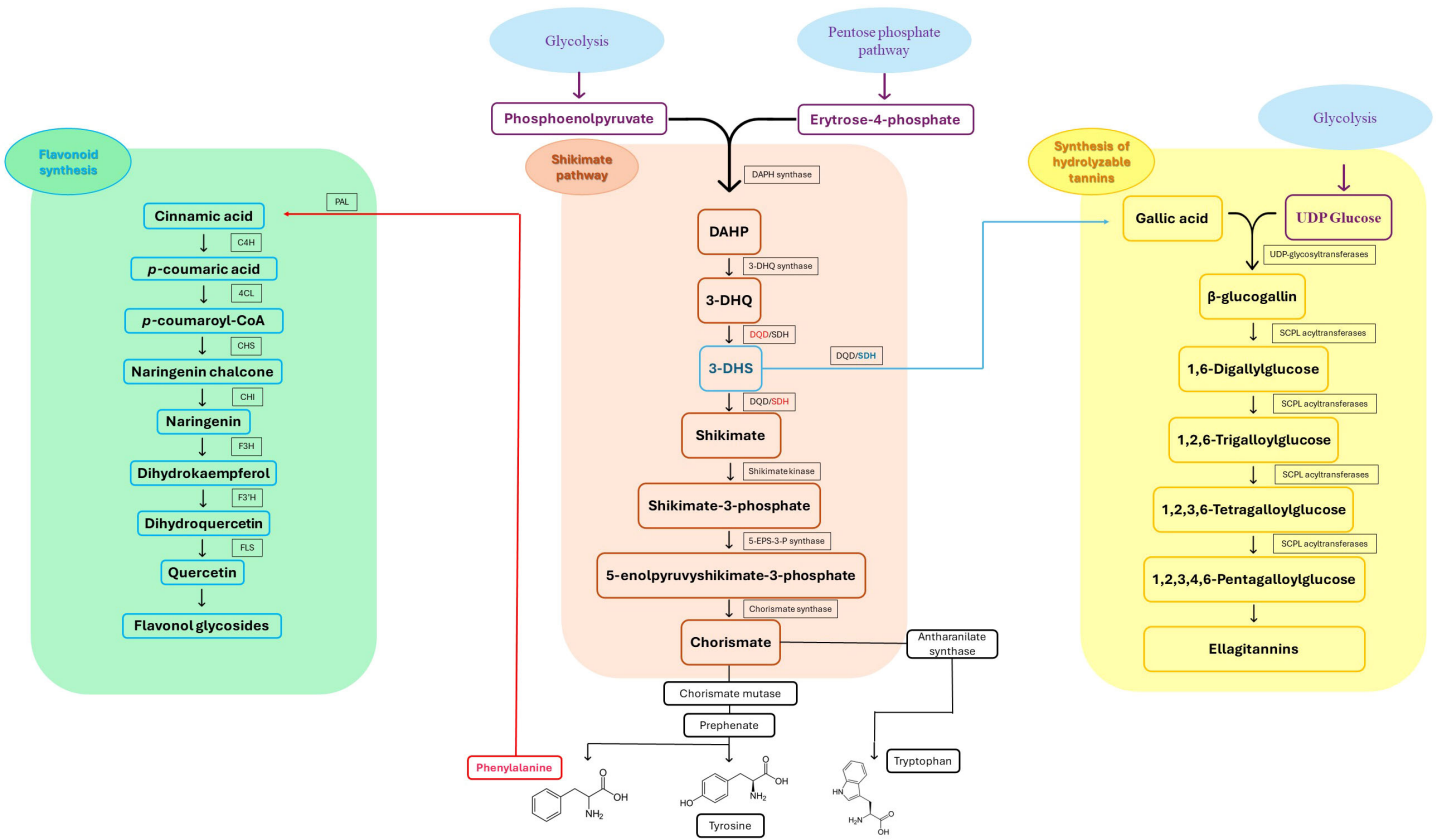
Integrated biosynthetic pathways of major phenolic compounds in *Eucalyptus* spp. The diagram connects the central Shikimate pathway (center) with specific downstream branches. Key metabolic precursors are text-colored to indicate bifurcation points: 3-Dehydroshikimate (3-DHS) (blue) and Phenylalanine (red). (Center, Shikimate pathway): The bifunctional enzyme dehydroquinate dehydratase/shikimate dehydrogenase (DQD/SDH) drives the central flux. Its DQD domain (red) catalyzes the dehydration of 3-DHQ to 3-DHS, while the SDH domain (red) performs the canonical reduction of 3-DHS to Shikimate for aromatic amino acid synthesis. (Left, Green panel): The Flavonoid biosynthesis pathway starts from Phenylalanine. Sequential action of enzymes like Phenylalanine ammonia-lyase (PAL) and Chalcone synthase (CHS) leads to Flavonol glycosides (e.g., quercetin-glucuronide), highly abundant in *Eucalyptus* leaves (Dos Santos Ferreira et al., 2016). (Right, Yellow panel): The Hydrolyzable tannin biosynthesis pathway diverges at 3-DHS. Specific *Eucalyptus* isoforms EcDQD/SDH2/3 exhibit an alternative SDH activity (blue) that converts 3-DHS directly into Gallic acid. This is subsequently esterified by UDP-glycosyltransferases (e.g., UGT84A) to form β-glucogallin, the precursor for Ellagitannins (Tahara et al., 2018).

Overall, compound-level mapping of the *Eucalyptus* phenolic metabolome confirms that diversity is shaped by species identity, organ type, and lineage-specific biosynthetic constraints. These insights provide a rational framework for prioritizing species and tissues during the pharmaceutical and biotechnological valorization of *Eucalyptus* phenolic compounds. The distinct chemical signatures and dominant compounds associated with each plant organ are summarized graphically in **Figure 2**, highlighting both shared metabolic cores and organ-specific specializations.

## 4 Phenolic compounds: biosynthesis, regulation, and functional roles

### 4.1 Biosynthetic origins via the Shikimate pathway

**Figure 3:**
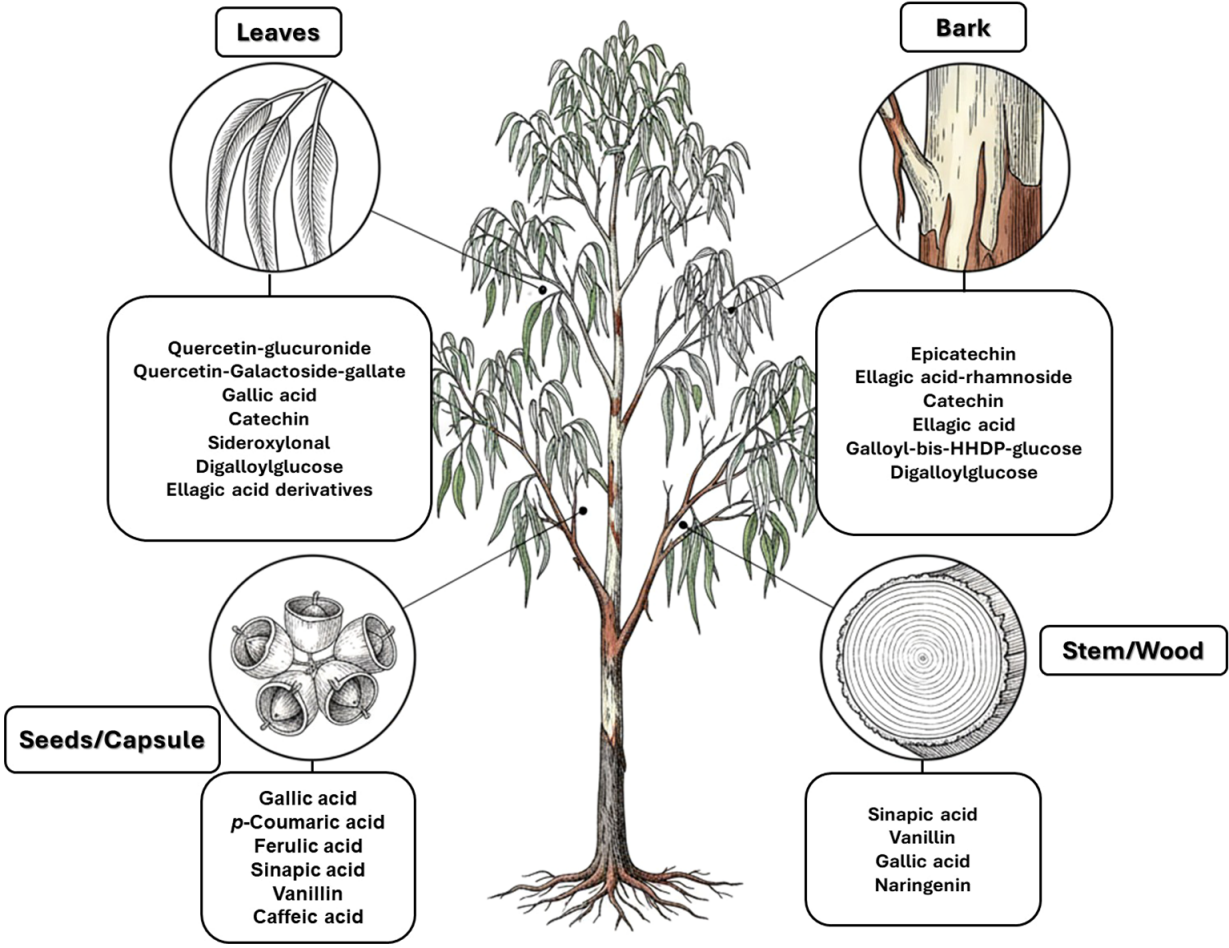
Organ-specific profiling of major phenolic constituents in *Eucalyptus* species. The diagram illustrates the differential accumulation of key phenolic metabolites across four distinct plant tissues. (Leaves): Characterized by a predominance of flavonol glycosides (e.g., quercetin-glucuronide) and hydroxybenzoic acids (gallic acid), along with the specific phloroglucinol derivative, sideroxylonal. (Bark): Distinguished by high concentrations of flavan-3-ols (particularly epicatechin) and complex hydrolyzable tannins such as ellagic acid-rhamnoside and galloyl-bis-HHDP-glucose. (Seeds/Capsules): Dominated by simple phenolic acids, including gallic, *p*-coumaric, and ferulic acids. (Stem/Wood): Defined by the presence of lignin-related phenolic acids and aldehydes, such as sinapic acid and vanillin. This spatial mapping emphasizes the chemical specialization of each tissue within the *Eucalyptus* metabolome.

Plant secondary metabolites fall into three major biosynthetic categories: phenolic compounds, encompassing phenolic acids, coumarins, stilbenes, lignans, flavonoids, tannins, and lignins; terpenoids, including volatiles, carotenoids, sterols, and glycosylated derivatives; and nitrogen-containing compounds such as alkaloids, cyanogenic glycosides, and glucosinolates (Ahlawat et al., 2024). These classes are derived from distinct yet interconnected metabolic routes linked to primary metabolism. Their integration with primary carbon metabolism, specifically glycolysis and the pentose phosphate pathway, is schematically illustrated in **Figure 3**. Among them, phenolic compounds are of particular importance: their aromatic ring systems bearing hydroxyl groups confer strong redox activity and diverse biological functions. They play central roles in defense, and protect plants against herbivores, pathogens, and UV radiation, while also participating in developmental signaling, structural reinforcement (e.g., lignification), and the modulation of stress responses. Collectively, these features enhance plant resilience across variable environments (Šamec et al., 2021; Ahlawat et al., 2024). The biosynthesis of phenolic compounds primarily originates from the shikimate pathway (**Figure 3**), which links carbohydrate metabolism to aromatic amino acid production: phenylalanine, tyrosine, and tryptophan (Maeda and Dudareva, 2012). Phenylalanine serves as the main substrate for the phenylpropanoid pathway, which produces flavonoids, anthocyanins, lignins, tannins, stilbenes, and coumarins (Maslennikova et al., 2023; Choi et al., 2023). Tyrosine contributes to specialized metabolites such as tocopherols (classified as terpenoids), cyanogenic glycosides, isoquinoline alkaloids, plastoquinone, and betalains. Tryptophan acts as a precursor for indole-based compounds, including auxins, indole alkaloids, and other specialized derivatives. This diversification of flux underscores the shikimate pathway’s central role in linking primary metabolism to diverse secondary metabolite biosynthesis.

### 4.2 Genetic, polyploid and nutrient-based regulation

In *Eucalyptus* species, phenolic compounds display striking structural diversity, ranging from low-molecular-weight phenolic acids to complex polyphenols such as lignins and hydrolysable tannins (Magina et al., 2024; Vieira et al., 2021). This heterogeneity underpins a wide range of functions, including abiotic stress tolerance, pathogen defense, and cell wall reinforcement. One notable case is *E. camaldulensis*, which exhibits enhanced tolerance to aluminum toxicity, a common limitation in acidic soils, via biosynthesis of oenothein B, a hydrolysable tannin with metal-chelating properties (Tahara et al., 2018). Crucially, recent biochemical characterization has elucidated that this formation of gallate from 3-dehydroshikimate is catalyzed by two specific NADP+ dependent isoenzymes, EcDQD/SDH2 and EcDQD/SDH3, effectively linking the central shikimate pathway with downstream polyphenol biosynthesis (Tahara et al., 2020). This bifunctional enzymatic architecture facilitates efficient flux toward gallate and illustrates the evolved biochemical strategies of *E. camaldulensis* for coping with edaphic stress.

Beyond detoxification, phenolics also reinforce structural and physiological integrity through core biochemical processes. Lignin, a complex phenolic polymer, strengthens secondary cell walls, supports long-distance water transport, and provides a barrier against biotic stressors (Ma, 2024). In *Eucalyptus*, lignin functions are particularly vital, enabling adaptation to drought, salinity, and mechanical injury (Keret et al., 2024). Lignin biosynthesis is tightly controlled, with multiple regulatory nodes modulating deposition rate, monomer composition, and tissue distribution in response to developmental and stress-related cues (Ziebell et al., 2016; Choi et al., 2023). This plasticity allows *Eucalyptus* species to dynamically adjust cell wall architecture under changing ecological pressures. Genetic regulation of this pathway is well-illustrated by polyploidy effects; for instance, triploid *E. urophylla* displays significantly lower lignin content, associated with the coordinated transcriptional downregulation of key lignin-biosynthetic regulators, including MYB52, MYB42, and NAC076 (Xu et al., 2023). Such regulatory control has also been exploited for bioenergy. Targeted genetic modification, particularly RNA interference (RNAi), has been used to downregulate lignin biosynthetic genes in *Eucalyptus* hybrids, thereby improving cell wall digestibility and reducing resistance during biomass conversion (Yuan et al., 2021). Lower lignin content enhances saccharification efficiency, decreases pretreatment requirements, and increases viability of *Eucalyptus* as a lignocellulosic feedstock. This capacity to reprogram phenolic pathways highlights the genus’s dual value in ecological resilience and industrial biotechnology (Ziebell et al., 2016).

Genetic regulation of phenolic metabolism in *Eucalyptus* has also been explored in the context of polyploidy. Xu et al. (2023) reported that triploid *E. urophylla* individuals display significantly lower lignin content than diploids. This reduction correlated with altered accumulation of lignin intermediates such as coniferaldehyde, p-coumaryl alcohol, and sinapaldehyde, indicating shifts in monolignol biosynthetic flux. Metabolomic profiling further showed transcriptional downregulation of key lignin regulators, including *MYB52*, *MYB42*, and *NAC076*. These results suggest that polyploidization can modulate lignin biosynthesis at both metabolic and transcriptional levels. Genome duplication may thus provide a tool for tailoring secondary metabolite profiles, especially in species where lignin content directly impacts biomass quality and processing efficiency.

In parallel, nutrient status has emerged as a critical determinant of phenolic pathway regulation. Zeng et al. (2024) demonstrated that nitrogen specifically modulates anthocyanin biosynthesis under nutrient limitation. In *Eucalyptus* seedlings, nitrogen supplementation (but not phosphate or potassium) suppressed anthocyanin accumulation normally induced by macronutrient deprivation. Transcriptomic data linked this suppression to the downregulation of genes encoding the MYB–bHLH–WDR transcriptional complex, particularly members of the *EgrMYB113* family. Functional validation through overexpression in *Eucalyptus* hairy roots confirmed that *EgrMYB113* activates the anthocyanin biosynthetic program, positioning it as a central regulatory node responsive to nitrogen status. Similar phenotypes were reported in *Arabidopsis myb113* mutants, where nitrogen deficiency failed to induce anthocyanins (Xie et al., 2016; Zeng et al., 2024), indicating evolutionary conservation of MYB113-like regulators across angiosperms.

These results reinforce the view that phenolic biosynthesis in *Eucalyptus* is governed by multilayered control, integrating genome architecture, nutrient sensing, and transcriptional regulation. This dynamic framework allows the plant to fine-tune phenolic accumulation in response to both developmental and environmental cues. Understanding these mechanisms is not only vital for dissecting woody perennial stress strategies but also pivotal for metabolic engineering aimed at boosting phenolic yields for industrial and pharmaceutical applications.

### 4.3 Phenolic responses to biotic stress

Phenolic compounds are equally central to pathogen defense, mediating immune responses and constraining disease spread (Saini et al., 2024). Pathogen attack typically triggers a rapid burst of ROS, damaging membranes, proteins, and DNA while simultaneously initiating defense signaling. In turn, plants induce protective mechanisms, including phenolic biosynthesis and activation of phenol-metabolizing enzymes (Kaur and Kaur, 2022). These compounds function as antioxidants quenching ROS, antimicrobials, lignin precursors for cell wall strengthening, and modulators of defense cascades. Complementary strategies involve tannin accumulation, phytoalexin synthesis, and cell wall remodeling. Lipophilic antioxidants such as carotenoids also contribute to ROS detoxification at the membrane level.

In *Eucalyptus*, phenolic metabolism is a decisive factor in tolerance. Sekiya et al. (2021), found that infection with *Austropuccinia psidii* (*Eucalyptus* rust) induced dynamic changes in phenolic profiles, including flavonoids, phenolic acids, and lignin precursors. In resistant genotypes, phenolic levels were initially suppressed but rose markedly after 12 hours postinoculation (hpi), aligning with defense establishment. By contrast, susceptible genotypes exhibited early, uncoordinated overaccumulation followed by strong suppression at 24 hpi, suggesting metabolic misregulation and weakened defense. These observations highlight the importance of timing in phenolic-mediated immunity.

Comparable patterns were reported by Xiaohui et al. (2022) during *Ralstonia solanacearum* infection of *E. urophylla*. Metabolomic and transcriptomic profiling revealed stage-specific accumulation of flavonoids and phenylpropanoids, indicating inducible rather than constitutive expression. Distinct metabolite signatures correlated with symptom progression, identifying potential biomarkers for early detection and resilience. This supports the notion that phenolics act not only as structural and antioxidant agents but also as integral components of inducible defense programs.

Together, these studies emphasize the pivotal role of phenolic metabolism in *Eucalyptus*–pathogen interactions. The ability to modulate biosynthesis spatially and temporally is central to constraining pathogen growth and preserving homeostasis under biotic stress. Moreover, coupling metabolite profiling with transcriptomic data provides a powerful approach to dissect tolerant mechanisms and guide the breeding of resilient genotypes. Overall, these findings underscore the regulatory complexity of phenolic metabolism in *Eucalyptus*, shaped by polyploidy, nutrient availability, and pathogen responses. Phenolics represent strategic targets for improving biomass quality, disease resistance, and industrial performance. Future work should address how abiotic stresses such as drought, heat, and salinity influence phenolic production and allocation. Expanding this knowledge could unlock the potential of phenolics as mediators of resilience and modulators of plant–environment interactions, supporting the development of *Eucalyptus* genotypes with enhanced adaptability and optimized metabolic profiles.

## 5 Applications of *Eucalyptus* phenolic compounds

Phenolic compounds have attracted considerable interest because of their strong antioxidant, antimicrobial, and anti-inflammatory properties, which support their use in pharmaceuticals, functional foods, and natural preservatives (Mihaylova et al., 2024; Zhang et al., 2022). Within this group, extracts from *Eucalyptus* species display a particularly broad spectrum of bioactivities, owing to their complex mixtures of flavonoids, tannins, and phenolic acids. These properties position *Eucalyptus*-derived phenolics as promising candidates for therapeutic development and sustainable industrial applications, including antimicrobial formulations, wound-healing agents, biodegradable antioxidants, and environmentally friendly preservatives (Shah et al., 2023; Pinto et al., 2023).

### 5.1 Phenolic compounds as potential pharmaceutical agents

Phenolic compounds have considerable pharmacological potential to prevent and manage complex chronic diseases such as cancer, neurodegenerative disorders (including Alzheimer’s disease), and metabolic syndromes like diabetes (Caruso et al., 2022; Khan et al., 2019; Yuan et al., 2022; Zhang et al., 2022). Their effects are primarily linked to redox-modulation, since phenolics can neutralize ROS and reduce oxidative damage to nucleic acids, proteins, and membrane lipids. This is important since ROS-induced damage is a cause of cellular dysfunction and disease progression (Durazzo et al., 2019; Mark et al., 2019). Specific phenolic acids such as ellagic acid, gallic acid, p-coumaric acid, and sinapic acid have been extensively investigated for their effects on ROS levels. In particular, p-coumaric acid and its derivatives display wide-ranging pharmacological effects, including antioxidant, antimicrobial, and anticancer activity, supporting their value as multipurpose therapeutic agents (Kaur and Kaur, 2022).

Ellagic acid has been reported to improve muscle endurance by promoting fiber-type transformation and stimulating mitochondrial biogenesis and function (Li et al., 2022). It also reduces muscle atrophy in diabetic models by alleviating mitochondrial dysfunction, endoplasmic reticulum stress, and apoptotic signaling (Liu et al., 2022). Similarly, p-coumaric acid contributes to ocular health by elevating reduced glutathione levels in the lens during early estrogen deficiency, potentially delaying cataract onset (Zych et al., 2019). Sinapic acid has also been associated with enhanced bone formation, indicating possible use in preventing or treating bone loss disorders (Balagangadharan et al., 2019).

*Eucalyptus* has long been used in traditional medicine as an antiseptic and antimicrobial remedy, especially for respiratory tract infections including colds, influenza, sore throats, and pneumonia (Moges et al., 2024; Sánchez-Loredo et al., 2024). Pharmacological studies have since confirmed these traditional roles by demonstrating direct antiviral activity in certain species. For example, *E. sideroxylon* leaf extracts reduced herpes simplex virus type 2 (HSV-2) replication by up to 87.65% *in vitro* (Okba et al., 2021). In addition, *E. microcorys* has shown notable anticancer effects: for example, aqueous leaf extracts and ethanolic fruit extracts have been found to inhibit the growth of glioblastoma, neuroblastoma, and lung and pancreatic cancer cell lines by >80% at a concentration of 100 μg/mL (Bhuyan et al., 2017). As a key agronomic application, beyond its antimicrobial heritage, *Eucalyptus* provides compounds with potential applications in virology and oncology.

Recent studies also highlight the anticancer promise of phenolic and triterpenoid compounds from *Eucalyptus* species. Triterpenoids have shown moderate inhibitory effects against several human cancer cell lines, including MDA-MB-231 (breast cancer), SGC-7901 (gastric adenocarcinoma), and HeLa (cervical cancer) (Wu et al., 2024). A comparative study by Liaudanskas et al. (2021) further assessed antioxidant and anticancer activities of phenolic-rich extracts from six medicinal plants, including *E. globulus*. Despite having the lowest TPC among the species tested, the acetone extract of *E. globulus* displayed the strongest anticancer effects against aggressive tumor cell lines (melanoma IGR39, glioblastoma U-87, and triple-negative breast cancer MDA-MB-231), as well as the highest antioxidant capacity in FRAP and other *in vitro* assays. These findings emphasize the notable bioefficacy of lipophilic phenolic compounds in *Eucalyptus* and underscore their potential as valuable agents for redox modulation and anticancer therapy. In addition, the lipophilic fraction of *E. camaldulensis* leaf extract (Lipo-Eucam) shows pharmacologically relevant properties, including antioxidant and anti-aggregation activity and cytotoxic effects against breast cancer cells, suggesting usefulness in addressing oxidative stress and neoplastic conditions (Huang et al., 2022). Overall, these data support continued exploration of lipophilic phytochemicals from *Eucalyptus* as candidates for integrated cancer therapy and redox-based interventions.

### 5.2 Antimicrobial activity and applications in food systems

The antimicrobial activity of plant-derived phenolic compounds represents a refined chemical defense system, integrating structural diversity with multitarget action to resist microbial invasion. These phytochemicals, ranging from simple phenolic acids to complex tannins and flavonoid polymers, exhibit broad antimicrobial properties shaped through coevolution with pathogens (Lobiuc et al., 2023; Sánchez-Loredo et al., 2024). Their effectiveness arises from physicochemical features that enable interactions with membranes, proteins, nucleic acids, and metal ions, thereby conferring activity even against multidrug-resistant strains (Zhang et al., 2022; Nguyen et al., 2023).

Phenolic acids are among the simplest yet most fundamental members of this defense system. Hydroxybenzoic acids (e.g., gallic, protocatechuic, vanillic) and hydroxycinnamic acids (e.g., caffeic, ferulic, p-coumaric) act as both antimicrobial agents and biosynthetic precursors (Ordoñez et al., 2022). In *Eucalyptus* species, these acids often occur at high concentrations. For example, ultrasonic ethanol extracts of *E. globulus* contained chlorogenic acid at 342.14 µg/mL and rosmarinic acid at 36.39 µg/mL (Sukhikh et al., 2022). Their antimicrobial activity involves dissipation of membrane potential through protonophoric action and the induction of oxidative stress via redox-cycling mechanisms (Tajfiroozeh et al., 2023). Structure–activity relationships further modulate potency; for instance, methoxy substitutions on the phenolic ring (as in ferulic acid) increase lipophilicity and facilitate membrane permeation, a feature particularly relevant for overcoming the outer-membrane barrier of Gram-negative bacteria (Zhang et al., 2019).

Flavonoids provide an even more elaborate antimicrobial arsenal, with their C6-C3-C6 framework allowing structural variations that support multiple modes of action (Górniak et al., 2019). *Eucalyptus* species, particularly *E. globulus* and *Corymbia torelliana*, are rich in flavonols such as quercetin, kaempferol, and myricetin (Al-Hadid et al., 2022; Sukhikh et al., 2022). These compounds intercalate with microbial DNA and chelate essential metals. For instance, quercetin-3D-glycoside, detected at 1,703.30 µg/mL in *E. globulus* extracts, inhibits *Pseudomonas aeruginosa* and *Candida albicans*, producing inhibition zones up to 15.5 mm (Sukhikh et al., 2022). B-ring dihydroxylation enhances antimicrobial potency by allowing hydrogen-bonding interactions with microbial enzymes. Paradoxically, this same catechol motif can also increase recognition by efflux pumps, which may in turn modify intracellular accumulation dynamics (Puljula et al., 2020).

Polyphenols such as ellagitannins also contribute to *Eucalyptus* antimicrobial defense. Compounds including pedunculagin and castalagin act through iron chelation and protein precipitation (Al-Harbi et al., 2017; Puljula et al., 2020). Ellagitannins from *E. camaldulensis* have shown notable antimicrobial activity and are under study for food preservation (Sánchez-Loredo et al., 2024). In addition to depriving microbes of iron, they may trigger Fenton chemistry-mediated oxidative stress (Zhang et al., 2022).

Other phenolic compounds, although less abundant, also strengthen the antimicrobial repertoire of *Eucalyptus*. Their potential should not be overlooked, as they may provide highly specific or synergistic effects. Stilbenes, for example, including resveratrol analogs, have been shown to inhibit quorum sensing in diverse bacterial systems (Tan et al., 2020). Although direct evidence for *Eucalyptus*-derived stilbenoids is limited, structural parallels suggest similar anti-virulence mechanisms, warranting targeted investigation. Furthermore, formyl-phloroglucinol meroterpenoids (FPMs), a unique class of phenolic–terpenoid hybrids confined to *Eucalyptus*, have recently been reported to exhibit antimicrobial activity. Wei et al. (2025), isolated eurobusone E and eurobusone F from *E. globulus* fruits, both of which showed moderate activity against *Staphylococcus aureus* (MIC = 16 µg/mL). These molecules retain a phloroglucinol scaffold fused to terpenoid moieties, and their hybrid architecture likely supports multiple mechanisms of action, including membrane destabilization and oxidative stress induction.

The antimicrobial phenolics of *Eucalyptus* are distributed according to tissue type and developmental stage. Young leaves accumulate small molecules such as gallic acid, whereas mature tissues are enriched in tannins and flavonoids that provide long-term protection (Sukhikh et al., 2022; Nobakht et al., 2017). This spatial and temporal regulation reflects an optimized metabolic strategy and generates a layered defense.

Advances in extraction techniques have further expanded practical applications. Ultrasound-assisted extraction improves phenolic recovery and preserves activity, with optimal conditions (25 min at 32 °C) producing extracts with strong antimicrobial effects (Sukhikh et al., 2022). Such extracts are being incorporated into food packaging systems for controlled release, maintaining > 90% inhibition of *Listeria monocytogenes* (Mokhtar et al., 2021).

In pharmacological settings, *Eucalyptus* phenolics can act synergistically with antibiotics. Flavonoid-rich extracts enhance antibiotic activity by disrupting membranes and possibly inhibiting efflux pumps (Zhang et al., 2019; Al-Hadid et al., 2022). Structure–activity relationship (SAR) analyses have identified specific moieties (including the 4-keto group and B-ring catechol motif) as major determinants of antimicrobial potency. These structural insights provide rational targets for synthetic modification and the development of optimized phenolic-based antimicrobial agents (Tajfiroozeh et al., 2023). Encapsulation technologies, including chitosan-based nanoparticles, are also being tested to stabilize phenolics and improve targeted delivery under physiological conditions (Santo et al. 2021). These innovations open opportunities for hybrid molecules that couple *Eucalyptus* phenolic scaffolds with conventional antibiotic pharmacophores, a strategy with strong promise for next-generation antimicrobials.

Overall, *Eucalyptus* phenolics represent a multiscale antimicrobial strategy. Their structural diversity, ecological specificity, and broad efficacy provide a strong foundation for developing sustainable interventions against microbial threats. This broad-spectrum antimicrobial and antioxidant capacity positions *Eucalyptus*-derived phenolics as compelling candidates for food preservation and active packaging technologies. Although most work on *Eucalyptus* in food systems has focused on essential oil components, yogurt fortified with 0.9% *E. camaldulensis* essential oil displayed significantly higher TPC and antioxidant capacity than controls, along with marked antibacterial effects against *Salmonella typhimurium* and *Escherichia coli* (Hamed et al., 2021). Molecular analysis linked part of this bioactivity to water-soluble phenolic constituents, emphasizing the potential of hydrophilic *Eucalyptus* phenolics in dairy formulations. Further optimization of extraction and formulation approaches could broaden their use across diverse food systems.

Phenolic acids such as p-coumaric acid and vanillin demonstrate multifunctionality in food applications, acting not only as preservatives but also as cross-linkers for edible films, flavor modulators, and antimicrobial coatings (Malik and Dhiman, 2022). Likewise, bark extracts of *E. globulus* have been used to produce phenolic-rich natural dyes (66.83–534.80 µg GAE/100 g DW) with high nutritional value and no detectable toxicity in murine models, supporting potential roles in food coloration and bioactive enhancement (Naseer et al., 2019).

Leaf phenolics from *Eucalyptus* also display broad-spectrum antimicrobial activity against foodborne pathogens. Ethanol extracts of *E. camaldulensis* leaves inhibited *Listeria monocytogenes*, *Staphylococcus aureus*, and *Bacillus cereus* (Nwabor et al., 2021). Among individual constituents, tellimagrandin I (an ellagitannin from *E. camaldulensis*) showed potent inhibition of *S. aureus* and *E. coli*, likely via membrane disruption and iron chelation (Puljula et al., 2020; Salih et al., 2021). Pedunculagin, another ellagitannin found in *E. camaldulensis*, has demonstrated anti-hemolytic and bacteriostatic effects in other plant systems such as *Pimenta dioica* (Al-Harbi et al., 2017), suggesting conserved activity relevant to *Eucalyptus*-based foods. Vescalagin, a structurally related ellagitannin, has displayed inhibitory action against a broader range of pathogens, including *L. monocytogenes, Salmonella enteritidis, B. cereus, E. coli*, and *S. aureus* (Fidelis et al., 2020; Sánchez-Loredo et al., 2024), further supporting the notion that *Eucalyptus* ellagitannins may be natural preservatives.

Beyond direct incorporation into foods, *Eucalyptus*-derived phenolics have also been studied in active packaging. Ghoshal and Singh (2020) produced nanocomposite films from starch and nanocellulose reinforced with 4% *E. globulus* leaf extract. These films exhibited improved barrier and mechanical properties as well as strong antioxidant and antimicrobial effects. They reduced microbial loads of *E. coli, L. monocytogenes, S. typhimurium*, and *Penicillium* spp. on fresh grapes during storage at both 25 °C and 4 °C over 7 to 28 days. Incorporating phenolic-rich *Eucalyptus* extracts into biodegradable matrices therefore offers a promising method to extend shelf life and enhance microbiological safety of perishables.

Recent advances have broadened the technological applications of *Eucalyptus* phenolics. For example, silver nanoparticles (AgNPs) synthesized using aqueous *E. globulus* leaf extract under microwave irradiation formed stable colloids with strong antimicrobial and antibiofilm activity against clinical strains including MRSA, *E. coli*, and *P. aeruginosa* (Ali et al., 2015). The capping of nanoparticles by phenolic and flavonoid moieties contributed to both colloidal stability and biological reactivity, suggesting applications in food-contact materials or biosensors.

Balčiūnaitienė et al. (2022) further reported that ethanolic extracts of *E. globulus*, containing ~29.9 mg GAE/g of phenolics, retained significant antioxidant capacity (ABTS and FRAP assays) and showed antimicrobial effects when combined with *Salvia officinalis* in phytosomal formulations. These vesicles inhibited *S. aureus, P. aeruginosa*, and *C. albicans*, demonstrating compatibility with emulsified delivery systems for nutraceutical and food applications. Moreover, wood vinegar derived from pyrolyzed *E. urograndis × E. grandis* biomass exhibited broad antimicrobial action against *B. subtilis, E. coli* O157:H7, and *Listeria monocytogenes*, with high contents of acetic and propionic acids (Gama et al., 2023). Its eco-friendly profile supports potential use as a bio-preservative or sanitizing agent in food processing.

Álvarez et al. (2021) compared six *Eucalyptus* species and found large interspecific variation in phenolic yield (235–523 μg GAE/g) and antioxidant activity. Notably, *E. globulus* exhibited particularly high anthocyanin and phenolic contents with strong radical scavenging potential, suggesting that targeted species selection and chemotypic profiling can optimize deployment of *Eucalyptus* resources in food systems.

Overall, phenolic compounds from *Eucalyptus* represent not only an ecologically adaptive chemical defense but also a renewable and versatile platform for enhancing food safety, preservation, and product value. Future work should prioritize nanoencapsulation technologies, synergistic use with conventional preservatives, and clear regulatory pathways to enable commercial adoption in food industries.

### 5.3 Other applications of *Eucalyptus* phenolic compounds

Phenolic compounds from *Eucalyptus* species display a wide range of bioactivities that extend well beyond their antioxidant potential. They are now being explored for agricultural, environmental, cosmetic, and biomedical applications owing to their allelopathic, antimicrobial, corrosion-inhibiting, and skin-protective properties.

In agriculture, phenolic-rich *Eucalyptus* extracts have shown strong allelopathic effects, suppressing germination and seedling growth in crops such as wheat (*Triticum durum*) and rapeseed (*Brassica rapa*). Andualem et al. (2024) demonstrated that both aqueous and methanolic leaf extracts of *E. camaldulensis* significantly reduced shoot and root growth, biomass, and yield in Ethiopian wheat cultivars in a dose-dependent manner. The methanolic extract proved particularly phytotoxic, impairing chlorophyll fluorescence and productivity across all cultivars tested. Similarly, Deng et al. (2024) reported that aqueous *Eucalyptus* extracts suppressed rapeseed germination yet enhanced root elongation at the seedling stage, highlighting stage-dependent and complex regulatory effects.

The allelopathic potential of *Eucalyptus*-derived phenolics also carries ecological significance. For example, Li et al. (2023) examined phenolic allelochemicals in *E. grandis* plantations across a chronosequence and observed that younger stands (especially 4 years old) contained elevated levels of flavonoids and phenolic acids such as ellagic acid, gallic acid, and quercetin. These compounds were negatively correlated with understory plant diversity, suggesting that chemical interference shapes community composition. Beyond agronomic usage, phenolic compounds from *Eucalyptus* also show potential for use in weed management. For example, Pinto et al. (2021) tested the herbicidal activity of postfire regenerated *E. globulus* leaves against *Portulaca oleracea*. Aqueous extracts of oven-dried leaves induced oxidative stress and inhibited growth in *P. Oleracea*; this may be a pathway for eco-friendly herbicidal applications. Reinforcing this potential, recent studies on aqueous extracts of *Eucalyptus* cinerea has demonstrated pronounced phytotoxicity against weed species, providing a plausible natural alternative to synthetic herbicides. This activity is attributed to their high content of allelopathic polyphenols, which exert inhibitory effects on germination and early seedling development (Grichi et al., 2025).

The antimicrobial potential of *Eucalyptus*-derived phenolics extends directly to crop protection and post-harvest preservation, providing sustainable alternatives to synthetic pesticides (Jiménez-Reyes et al., 2019). Extracts enriched in phenolic acids, flavonoids, and tannins exhibit strong antifungal activity against major necrotrophic pathogens. For example, phenolic-rich bark extracts of *E. camaldulensis* suppressed mycelial growth of *Fusarium culmorum* and *Botrytis cinerea* by more than 40% in wood bioassays (Abdelkhalek et al., 2020), while extracts from *E. globulus* demonstrated inhibitory effects on wood-rot basidiomycetes such as *Trametes versicolor* (González et al., 2017).

Furthermore, these compounds show considerable promise for managing post-harvest diseases, including blue mold caused by *Penicillium expansum* in pome fruits (Luciano-Rosario et al., 2020; Lv et al., 2024). Their bioactivity is underpinned by a multitarget mode of action, including disruption of fungal cell membranes, inhibition of key metabolic enzymes, and chelation of essential metal ions (Silva-Beltran et al., 2023). Such polypharmacology is particularly advantageous for resistance management, as structurally diverse phenolic mixtures are less likely to select for resistant pathogen strains than single-site fungicides.

Beyond fungal pathogens, *Eucalyptus* phenolics also exhibit substantial antibacterial potential. Extracts of *E. cinerea* have demonstrated activity against *Agrobacterium tumefaciens*, the causal agent of crown gall disease (Kahla et al., 2017). Expanding on bacterial control, aqueous leaf extracts of *E. globulus* display differential inhibitory effects against major tomato pathogens, including *Xanthomonas vesicatoria* and *Clavibacter michiganensis* (Pinto et al., 2023). Collectively, these findings underscore the versatility of *Eucalyptus*-derived phenolics for the management of economically significant phytobacterioses, complementing their well-documented efficacy against fungal pathogens. In postharvest systems, phenolic compounds from *Eucalyptus* leaf extracts have been shown to mitigate oxidative damage and extend shelf life. Haider et al. (2022) found that strawberries treated with 30% *E. globulus* leaf extract exhibited reduced fungal decay and enhanced antioxidant enzyme activity during storage, leading to improvements in firmness, flavor, and consumer acceptance.

These compounds are also relevant to cosmetic and dermatological applications. Almeida et al. (2022) developed bacterial nanocellulose membranes infused with aqueous *E. globulus* extract for anti-aging purposes. These membranes displayed dose-dependent antioxidant activity, stability over time, and noncytotoxicity in human skin and fibroblast cells, significantly lowering markers of cellular senescence. A broader safety evaluation by Becker et al. (2023) confirmed that *E. globulus*-derived ingredients are safe for cosmetic use when formulated to avoid sensitization.

Biomedical research has also pointed to new uses. Ellagitannins extracted from *E. camaldulensis* with ultrasound–microwave hybrid technology exhibited strong antioxidant activity (DPPH IC_50_ = 371.13 mg/L) and inhibited *Giardia lamblia* growth by up to 80% after 48 hours, suggesting antiparasitic potential (Sánchez-Loredo et al., 2025).

In environmental applications, *Eucalyptus* phenolics have demonstrated efficacy as green corrosion inhibitors. Aslam et al. (2022) evaluated ethanolic bark extracts of *Eucalyptus* on mild steel exposed to 5% HCl. Using gravimetric analysis, electrochemical impedance spectroscopy, and potentiodynamic polarization (PDP), they found inhibition efficiency of 98.2% at 900 ppm and 30 °C. The extract, rich in flavonoids, saponins, alkaloids, and terpenoids, acted through adsorption of phytoconstituents onto the steel surface, following the Langmuir adsorption isotherm. This suggested a monolayer with mixed physical and chemical adsorption. Scanning electron microscopy further confirmed a protective film that reduced acid-induced damage. These findings emphasize *Eucalyptus* bark extracts as an eco-friendly alternative to synthetic corrosion inhibitors.

Overall, the diverse activities of *Eucalyptus*-derived phenolics highlight a variety of potential use cases across sustainable agriculture, ecological restoration, postharvest preservation, cosmetic development, and environmental protection.

## 6 Prospects and challenges for the use of phenolic compounds from *Eucalyptus*

Phenolic compounds from *Eucalyptus* represent promising avenues for bioactive innovation, but their deployment at scale is still limited by technical, biological, and regulatory barriers. Contemporary research on plant phenolics emphasizes not only potency but also extractability, bioavailability, and chemotypic standardization. In comparison with model or cultivated species (Zhang et al., 2022; Ahlawat et al., 2024), these aspects remain poorly characterized in *Eucalyptus*. This variability underscores a deeper conceptual limitation: *Eucalyptus* phenolics are frequently interpreted as static chemical inventories rather than dynamic, plastic traits shaped by genotype, environment, and their interactions. In the absence of frameworks that explicitly link metabolomic profiles to adaptive function, many datasets risk remaining descriptive catalogs with limited explanatory power. Developing causal models that integrate metabolic allocation with ecological strategies is therefore essential to advance from pattern recognition to mechanistic understanding.

A critical barrier that has not been sufficiently addressed is the lack of mechanistic integration between ecological observations and biochemical data. Most reports emphasize quantitative yields or compound inventories, yet very few studies connect these profiles to gene expression dynamics, enzymatic fluxes, or adaptive ecological strategies. Without this integration, phenolic datasets remain largely descriptive and fail to explain why certain species or tissues invest disproportionately in specific phenolic classes. A more mechanistic approach, combining high-resolution metabolomics with transcriptomic and functional validation, is necessary to uncover the evolutionary logic and the metabolic trade-offs that underlie phenolic diversity in *Eucalyptus*.

A major bottleneck limiting the translational value of current research is the absence of methodological standardization. As noted in Section 2.1, the heterogeneity of analytical platforms (spanning low-resolution colorimetric assays to high-resolution QTOF-MS) introduces substantial variability in reported chemotypes. For instance, studies employing UPLC-QTOF-MS (e.g., Huang et al., 2022; Romano et al., 2025) detect a markedly broader and more structurally resolved metabolite spectrum than those using conventional HPLC-UV (e.g., Abdelkhalek et al., 2020). This methodological divergence not only hinders cross-study comparability but also obscures the true magnitude of *Eucalyptus* chemodiversity, rendering robust meta-analyses nearly unattainable. Unlike *Camellia sinensis* or *Vitis vinifera*, where phenolic composition is genetically mapped and agronomically stabilized (Maritim et al., 2023; Ahn et al., 2023), *Eucalyptus* shows pronounced intra- and interspecific variability. This reflects both its genetic diversity and the influence of environmental and developmental factors on phenolic biosynthesis. For example, leaf TPC in *E. globulus* has been reported at 88.34 mg/g with 100% methanol extraction (Santos et al., 2011), whereas similar protocols yielded only 26.88 mg/g in other studies (Álvarez et al., 2021). Such variability, driven in part by genotype × environment interactions (e.g., arid vs. mesic conditions in *E. camaldulensis*), complicates reproducibility and translation into industrial applications. Developing species–tissue metabolomic atlases using LC-MS/MS and NMR is therefore essential, particularly to identify high-value chemotypes such as *E. marginata*, which uniquely accumulates rosmarinic acid and apigenin derivatives (Djebbi et al., 2024; Hasni et al., 2021). Despite the value of these metabolic atlases, most current reports remain case-by-case and rarely adopt standardized protocols. The absence of coordinated cross-laboratory benchmarking means that datasets are not directly comparable, and meta-analyses are almost impossible. Addressing this requires multi-site, multi-species initiatives with harmonized extraction, quantification, and data-sharing pipelines; otherwise, the same descriptive problems will persist, only multiplied.

Advances in extraction technologies are encouraging. For example, microwave-assisted extraction (MAE) has increased TPC yields from *E. robusta* and *E. globulus* leaves and bark while reducing solvent use and processing time (Bhuyan et al., 2015; Tomasi et al., 2023). Natural deep eutectic solvents (NaDES) also show promise: betaine:ethylene glycol systems achieved polyphenol yields of 22.4%, outperforming 70% acetone (~13.2%) under comparable conditions (Jauregi et al. 2024). Hydrophilic NaDES are particularly effective for flavonoids, whereas hydrophobic systems (e.g., menthol:thymol) have been used for triterpenoids from *E. globulus* bark (Silva et al., 2020). However, obstacles remain. For example, solvent viscosity, selectivity, and recovery efficiency differ widely between systems; hybrid methods (e.g., ultrasound–microwave coupling) for ellagitannin extraction are underexplored; and scalability to biorefineries is still limited. A rational approach would be co-extraction of essential oils and phenolics from postdistillation biomass (stump wood, bark residues) to maximize yields while minimizing waste (Tomasi et al., 2023). Despite these advances, most extraction research remains proof-of-concept, with little connection to techno-economic reality. Few studies report energy balances, solvent recyclability, or integration into existing pulp/biorefinery pipelines. Without this dimension, claims of “green extraction” remain speculative. Future work should benchmark extraction methods against industrial metrics (cost, throughput, and co-product compatibility) rather than focusing exclusively on laboratory yields.

Bioavailability and delivery also present hurdles. Poorly soluble compounds such as ellagitannins, dimeric flavonoids, and quercetin-glucuronide often show limited systemic activity. Encapsulation in phytosomes or lipid nanoparticles has improved delivery in other plants (e.g., *Curcuma longa*, *Camellia sinensis*), but such systems remain scarcely developed in *Eucalyptus* (Xie et al., 2016). Starch–nanocellulose films containing *E. globulus* extracts have demonstrated antioxidant and antimicrobial effects in food systems (Ghoshal and Singh, 2020), suggesting a platform for dermal or mucosal applications. Chitosan nanoparticles could also be explored for ellagitannin encapsulation, given their proven compatibility with polyphenols and biocompatibility (Al-Hadid et al., 2022). The translational bottleneck is not only solubility but also the absence of standardized pharmacological pipelines. Most assays stop at antioxidant capacity or *in vitro* cytotoxicity, endpoints that poorly predict therapeutic value. A next step is to implement structured ADME (Absorption, Distribution, Metabolism, and Excretion) and toxicology workflows, integrating animal models and early safety evaluation. Only by identifying lead compounds with demonstrable *in vivo* efficacy will *Eucalyptus* phenolics progress from academic curiosity to realistic clinical or industrial candidates.

From a regulatory perspective, most *Eucalyptus* phenolics lack Generally Recognized as Safe designation, limiting their adoption in food and cosmetics. Cytotoxicity studies in human dermal cells suggest good safety for aqueous bark extracts (Becker et al., 2023), yet systematic preclinical evaluations (e.g., oral toxicity in murine models) and clear regulatory pathways through FDA and EFSA are needed to enable commercial use. Ecological risks should also be considered. Allelopathic phenolics such as gallic and ellagic acids, released by *E. grandis* roots, reduce understory plant diversity and shift microbial communities (Li et al., 2023). Similar to juglone in *Juglans* spp., their safe agricultural application requires robust dose–response studies and soil mobility modeling. Preliminary thresholds (≤1% aqueous root extract) have been proposed for preemergence use, but field validation remains lacking.

Finally, although >103 phenolic compounds have been cataloged in *Eucalyptus*, documentation often lacks reproducibility and chemometric comparability. A coordinated metabolomic initiative integrating LC-MS/MS, GC-MS, and NMR across taxa such as *E. globulus*, *E. camaldulensis*, *E. maidenii*, and *E. marginata* would clarify biosynthetic trends and support selective breeding or metabolic engineering. Overall, making greater use of *Eucalyptus* phenolics requires moving beyond descriptive chemical inventories toward standardized extraction, advanced delivery systems, regulatory validation, and ecological safeguards. Integrative strategies linking omics-informed chemotype selection, green extraction, and coproduct recovery within low-emission biorefineries are essential to position *Eucalyptus* phenolics as next-generation bioactives in health, agriculture, and materials science.

## 7 Conclusions

*Eucalyptus* species represent a vast and underexploited reservoir of structurally diverse phenolic compounds that show compelling potential regarding a wide range of pharmaceutical, food, agricultural, and environmental applications. This review demonstrates that phenolic accumulation is highly dependent on species identity, tissue type, and ecological context. Overall, bark and stump wood are the most phenolic-rich tissues, but are often overlooked in valorization pipelines focusing on bioactive compounds. Detailed compound-level mapping reveals species- and organ-specific biosynthetic patterns, underscoring the need for direct chemical characterization rather than taxonomic extrapolation.

On the biochemical scale, phenolic biosynthesis in *Eucalyptus* is regulated by complex networks that integrate primary metabolism, nutrient status, polyploidy, and pathogen responses. These traits confer both metabolic flexibility and ecological resilience, but they also complicate phenolic standardization across genotypes and environments. Although numerous therapeutic and technological applications have been validated *in vitro* including antioxidant, antimicrobial, and anticancer properties, practical application remains limited by poor solubility, inconsistent extractability, and incomplete regulatory integration. Despite these hurdles, several high-value applications already demonstrate clear translational potential. *Eucalyptus* phenolics are particularly well positioned for real-world deployment as biopesticides in sustainable agriculture, as natural antioxidants for extending food shelf-life through active packaging technologies, and as green corrosion inhibitors for industrial infrastructure, each offering renewable, biologically derived alternatives to conventional synthetic additives.

However, several critical knowledge gaps still constrain broader deployment. Foremost among these is the absence of standardized metabolomic reporting, which limits cross-study comparability, alongside the incomplete elucidation of the molecular mechanisms driving the bioactivity of complex phenolic extracts. Addressing these limitations will require a shift from predominantly descriptive profiling toward integrative, mechanistic frameworks. Key priorities include: (i) establishing comprehensive metabolomic and genomic atlases to resolve species- and tissue-level chemodiversity; (ii) elucidating the genetic regulation of phenolic traits under abiotic and biotic stress to inform breeding and metabolic engineering strategies; and (iii) implementing rigorous field trials to validate efficacy and stability of these compounds in agricultural and environmental applications.

Overall, this metabolically rich genus offers not only a chemical basis for plant resilience and a renewable innovation platform for health, industry, and environmental sustainability.
