# Supplementary material for: Chemodiversity, biosynthetic regulation, and functional roles of Eucalyptus phenolics: applications and prospects
Source: Front Plant Sci. 2026 Jan 12;16:1733676. doi: 10.3389/fpls.2025.1733676 (PMC12832836; doi:10.3389/fpls.2025.1733676)
Supplement: Supplementary file 1 [file Table1.docx]

**Supplementary File 1**. Consolidated phenolic constituents in *Eucalyptus*. Data were compiled from peer-reviewed studies published between 2009 and 2025, covering multiple *Eucalyptus* species and plant tissues.

| Compound | *Eucalyptus*  species | Plant tissues | Content*  (mg/g dry weight) | Reference |
| --- | --- | --- | --- | --- |
| Quinic acid | *E. grandis* | Bark | 3.68 ± 0.16 | Santos et al. (2012) |
| Gallic acid | *E. grandis* | Bark | 10.61 ± 0.24 | Santos et al. (2012) |
| Protocatechuic acid | *E. grandis* | Bark | 1.83 ± 0.06 | Santos et al. (2012) |
| Methyl gallate | *E. grandis* | Bark | 1.54 ± 0.04 | Santos et al. (2012) |
| Catechin | *E. grandis* | Bark | 22.79 ± 0.43 | Santos et al. (2012) |
| Ellagic acid | *E. grandis* | Bark | 25.43 ± 0.39 | Santos et al. (2012) |
| Galloyl-bis-HHDP-glucose | *E. grandis* | Bark | 15.81 ± 0.19 | Santos et al. (2012) |
| Digalloylglucose | *E. grandis* | Bark | 14.75 ± 0.11 | Santos et al. (2012) |
| Epicatechin | *E. grandis* | Bark | 68.2 ± 0.67 | Santos et al. (2012) |
| Ellagic acid-rhamnoside | *E. grandis* | Bark | 47.32 ± 0.42 | Santos et al. (2012) |
| Isorhamnetin-rhamnoside | *E. grandis* | Bark | 1.17 ± 0.04 | Santos et al. (2012) |
| Quinic acid | *E. urograndis* | Bark | 7.54 ± 0.25 | Santos et al. (2012) |
| Gallic acid | *E. urograndis* | Bark | 15.84 ± 0.16 | Santos et al. (2012) |
| Protocatechuic acid | *E. urograndis* | Bark | 1.27 ± 0.06 | Santos et al. (2012) |
| Methyl gallate | *E. urograndis* | Bark | 2.46 ± 0.04 | Santos et al. (2012) |
| Catechin | *E. urograndis* | Bark | <LOD | Santos et al. (2012) |
| Ellagic acid | *E. urograndis* | Bark | 14.99 ± 0.21 | Santos et al. (2012) |
| Naringenin | *E. urograndis* | Bark | <LOD | Santos et al. (2012) |
| Galloyl-bis-HHDP-glucose | *E. urograndis* | Bark | 17.34 ± 0.3 | Santos et al. (2012) |
| Epicatechin | *E. urograndis* | Bark | 118.86 ± 0.71 | Santos et al. (2012) |
| Ellagic acid-rhamnoside | *E. urograndis* | Bark | 10.81 ± 0.15 | Santos et al. (2012) |
| Isorhamnetin-rhamnoside | *E. urograndis* | Bark | 1.33 ± 0.06 | Santos et al. (2012) |
| Quinic acid | *E. maidenii* | Bark | 5.74 ± 0.12 | Santos et al. (2012) |
| Gallic acid | *E. maidenii* | Bark | 7.95 ± 0.16 | Santos et al. (2012) |
| Protocatechuic acid | *E. maidenii* | Bark | 1.17 ± 0.04 | Santos et al. (2012) |
| Methyl gallate | *E. maidenii* | Bark | 1.59 ± 0.08 | Santos et al. (2012) |
| Catechin | *E. maidenii* | Bark | 34.44 ± 0.24 | Santos et al. (2012) |
| Chlorogenic acid | *E. maidenii* | Bark | 10.99 ± 0.29 | Santos et al. (2012) |
| Ellagic acid | *E. maidenii* | Bark | 8.72 ± 0.18 | Santos et al. (2012) |
| Taxifolin | *E. maidenii* | Bark | 4.91 ± 0.15 | Santos et al. (2012) |
| Quercetin-hexoside | *E. maidenii* | Bark | 2.18 ± 0.10 | Santos et al. (2012) |
| Quercetin | *E. maidenii* | Bark | 0.16 ± 0.01 | Santos et al. (2012) |
| Isorhamnetin | *E. maidenii* | Bark | 0.95 ± 0.04 | Santos et al. (2012) |
| Naringenin | *E. maidenii* | Bark | 0.87 ± 0.01 | Santos et al. (2012) |
| Galloyl-bis-HHDP-glucose | *E. maidenii* | Bark | 8.44 ± 0.37 | Santos et al. (2012) |
| Dihydroxy-isopropylchromone-hexoside | *E. maidenii* | Bark | 2.7 ± 0.09 | Santos et al. (2012) |
| Isorhamnetin-hexoside | *E. maidenii* | Bark | 0.72 ± 0.02 | Santos et al. (2012) |
| Ellagic acid-rhamnoside | *E. maidenii* | Bark | 3.13 ± 0.13 | Santos et al. (2012) |
| Methyl-ellagic acid-pentose | *E. maidenii* | Bark | 9.16 ± 0.39 | Santos et al. (2012) |
| Myricetin-rhamnoside | *E. maidenii* | Bark | 5.27 ± 0.17 | Santos et al. (2012) |
| Mearnsetin | *E. maidenii* | Bark | 0.15 ± 0.01 | Santos et al. (2012) |
| Mearnsetin-hexoside | *E. maidenii* | Bark | 0.25 ± 0.01 | Santos et al. (2012) |
| Eriodictyol | *E. maidenii* | Bark | 0.93 ± 0.04 | Santos et al. (2012) |
| Isorhamnetin | *E. maidenii* | Bark | 0.95 ± 0.04 | Santos et al. (2012) |
| Gallic acid | *E. tereticornis* | Leaves | 3.53 ± 0.13 | Chaudhry et al. (2023) |
| Naringenin | *E. tereticornis* | Leaves | 4.29 ± 0.12 | Chaudhry et al. (2023) |
| Sinapic acid | *E. tereticornis* | Leaves | 2.95 ± 0.02 | Chaudhry et al. (2023) |
| Caffeic acid | *E. tereticornis* | Leaves | 4.47 ± 0.12 | Chaudhry et al. (2023) |
| p-Hydroxy benzoic acid | *E. tereticornis* | Leaves | 1.43 ± 0.01 | Chaudhry et al. (2023) |
| p-coumaric acid | *E. tereticornis* | Leaves | 3.21 ± 0.11 | Chaudhry et al. (2023) |
| Ferulic acid | *E. tereticornis* | Leaves | 8.71 ± 0.23 | Chaudhry et al. (2023) |
| Gallic acid | *E. tereticornis* | Stem | 3.53 ± 0.12 | Chaudhry et al. (2023) |
| Naringenin | *E. tereticornis* | Stem | 2.1 ± 0.11 | Chaudhry et al. (2023) |
| Sinapic acid | *E. tereticornis* | Stem | 4.68 ± 0.11 | Chaudhry et al. (2023) |
| p-Hydroxy benzoic acid | *E. tereticornis* | Stem | 11.03 ± 1.02 | Chaudhry et al. (2023) |
| Vanillin | *E. tereticornis* | Stem | 3.83 ± 0.13 | Chaudhry et al. (2023) |
| Vanillic acid | *E. tereticornis* | Stem | 1.57 ± 0.09 | Chaudhry et al. (2023) |
| m-coumaric acid | *E. tereticornis* | Stem | 1.45 ± 0.04 | Chaudhry et al. (2023) |
| Gallic acid | *E. tereticornis* | Seed | 1.36 ± 0.05 | Chaudhry et al. (2023) |
| Naringenin | *E. tereticornis* | Seed | 4.53 ± 0.14 | Chaudhry et al. (2023) |
| Sinapic acid | *E. tereticornis* | Seed | 0.62 ± 0.01 | Chaudhry et al. (2023) |
| Caffeic acid | *E. tereticornis* | Seed | 6.49 ± 0.16 | Chaudhry et al. (2023) |
| p-coumaric acid | *E. tereticornis* | Seed | 16.66 ± 1.21 | Chaudhry et al. (2023) |
| Ferulic acid | *E. tereticornis* | Seed | 8.14 ± 1.11 | Chaudhry et al. (2023) |
| Catechin | *E. tereticornis* | Seed | 3.23 ± 0.12 | Chaudhry et al. (2023) |
| Gallic acid | *E. tereticornis* | Testa | 23.47 ± 1.25 | Chaudhry et al. (2023) |
| Naringenin | *E. tereticornis* | Testa | 3.1 ± 0.12 | Chaudhry et al. (2023) |
| Sinapic acid | *E. tereticornis* | Testa | 6.05 ± 0.31 | Chaudhry et al. (2023) |
| Caffeic acid | *E. tereticornis* | Testa | 1.15 ± 0.11 | Chaudhry et al. (2023) |
| p-Hydroxy benzoic acid | *E. tereticornis* | Testa | 1.43 ± 0.13 | Chaudhry et al. (2023) |
| Ferulic acid | *E. tereticornis* | Testa | 2.5 ± 0.17 | Chaudhry et al. (2023) |
| Vanillin | *E. tereticornis* | Testa | 5.71 ± 0.21 | Chaudhry et al. (2023) |
| Vanillic acid | *E. tereticornis* | Testa | 0.44 ± 0.01 | Chaudhry et al. (2023) |
| Chlorogenic acid | *E. tereticornis* | Testa | 0.8 ± 0.15 | Chaudhry et al. (2023) |
| Cinnamic acid | *E. tereticornis* | Testa | 1.61 ± 0.15 | Chaudhry et al. (2023) |
| Gallic acid | *E. globulus* | Leaves | 142 ± 9 | Romano et al. (2025) |
| Protocatechuic acid | *E. globulus* | Leaves | 14 ± 2 | Romano et al. (2025) |
| Methyl gallate | *E. globulus* | Leaves | 21 ± 2 | Romano et al. (2025) |
| Catechin | *E. globulus* | Leaves | 105 ± 4 | Romano et al. (2025) |
| Chlorogenic acid | *E. globulus* | Leaves | 17 ± 1 | Romano et al. (2025) |
| Digalloylglucose | *E. globulus* | Leaves | 60 ± 6 | Romano et al. (2025) |
| Digalloylglucose isomer | *E. globulus* | Leaves | 54 ± 15 | Romano et al. (2025) |
| HHDP-galloylglucose | *E. globulus* | Leaves | 15 ± 4 | Romano et al. (2025) |
| Tellimagrandin | *E. globulus* | Leaves | 45 ± 5 | Romano et al. (2025) |
| Trigalloylglucose | *E. globulus* | Leaves | 21 ± 4 | Romano et al. (2025) |
| Tetragalloylglucose | *E. globulus* | Leaves | 14 ± 2 | Romano et al. (2025) |
| Methylphloroglucinol-digalloyl glucose | *E. globulus* | Leaves | 30 ± 9 | Romano et al. (2025) |
| Quercetin-galactoside-gallate | *E. globulus* | Leaves | 148.2 ± 0.3 | Romano et al. (2025) |
| Isorhamnetin-hexoside | *E. globulus* | Leaves | 41 ± 1 | Romano et al. (2025) |
| Quercetin-glucuronide | *E. globulus* | Leaves | 407 ± 15 | Romano et al. (2025) |
| Methyl-ellagic acid-pentose | *E. globulus* | Leaves | 44 ± 5 | Romano et al. (2025) |
| Quercetin-hexoside | *E. globulus* | Leaves | 16 ± 1 | Romano et al. (2025) |
| Isorhamnetin-rhamnoside | *E. globulus* | Leaves | 24 ± 4 | Romano et al. (2025) |
| Quercetin | *E. globulus* | Leaves | 31 ± 2 | Romano et al. (2025) |
| Dimethyl-hesperetin | *E. globulus* | Leaves | 7 ± 1 | Romano et al. (2025) |
| Naringenin | *E. globulus* | Leaves | 3.2 ± 0.7 | Romano et al. (2025) |
| Cypellocarpin C | *E. globulus* | Leaves | 5.7 ± 1.5 | Romano et al. (2025) |
| Ellagic acid | *E. globulus* | Leaves | 11.51 | Boulekbache‐Makhlouf et al. (2013) |
| Gallic acid | *E. globulus* | Leaves | 0.84 | Boulekbache‐Makhlouf et al. (2013) |
| Eucaglobulin | *E. globulus* | Leaves | 2.11 | Boulekbache‐Makhlouf et al. (2013) |
| Globulusin B | *E. globulus* | Leaves | 3.67 | Boulekbache‐Makhlouf et al. (2013) |
| Cypellocarpin C | *E. globulus* | Leaves | 0.47 | Boulekbache‐Makhlouf et al. (2013) |
| 3-O-methylellagic acid 3’-α-rhamnoside | *E. globulus* | Leaves | 0.58 | Boulekbache‐Makhlouf et al. (2013) |
| Methylellagic acid-acetylrhamnoside | *E. globulus* | Leaves | 0.25 | Boulekbache‐Makhlouf et al. (2013) |
| Methylellagic acid 3-O-pentoside | *E. globulus* | Leaves | 0.42 | Boulekbache‐Makhlouf et al. (2013) |
| Digalloylglucose | *E. globulus* | Leaves | 0.12 | Boulekbache‐Makhlouf et al. (2013) |
| Ellagic acid hexose | *E. globulus* | Leaves | 0.16 | Boulekbache‐Makhlouf et al. (2013) |
| Methylellagic acid hexose | *E. globulus* | Leaves | 7.08 | Boulekbache‐Makhlouf et al. (2013) |
| Glucoside of dimethylellagic acid | *E. globulus* | Leaves | 0.34 | Boulekbache‐Makhlouf et al. (2013) |
| Methylellagic acid | *E. globulus* | Leaves | 0.56 | Boulekbache‐Makhlouf et al. (2013) |
| Quercetin-3-O-glycoside | *E. globulus* | Leaves | 0.05 | Boulekbache‐Makhlouf et al. (2013) |
| Quercetin-3-O-β-D-glucuronide | *E. globulus* | Leaves | 2.65 | Boulekbache‐Makhlouf et al. (2013) |
| Quercetin-3-O-β-D-galactoside-6’’-O-gallate | *E. globulus* | Leaves | 0.07 | Boulekbache‐Makhlouf et al. (2013) |
| Quercetin-3-O-rhamnoside | *E. globulus* | Leaves | 4.39 | Boulekbache‐Makhlouf et al. (2013) |
| Cypellogine A/B | *E. globulus* | Leaves | 0.36 | Boulekbache‐Makhlouf et al. (2013) |
| Tetragalloylglucose | *E. globulus* | Leaves | 2.17 | Boulekbache‐Makhlouf et al. (2013) |
| Pentagalloylglucose | *E. globulus* | Leaves | 0.66 | Boulekbache‐Makhlouf et al. (2013) |
| Pedunculagin | *E. globulus* | Leaves | 0.61 | Boulekbache‐Makhlouf et al. (2013) |
| HHDP Galloyl glucose isomer | *E. globulus* | Leaves | 0.51 | Boulekbache‐Makhlouf et al. (2013) |
| TrisHHDP galloyl glucose isomer | *E. globulus* | Leaves | 0.04 | Boulekbache‐Makhlouf et al. (2013) |
| Oenothein B | *E. globulus* | Leaves | 3.01 | Boulekbache‐Makhlouf et al. (2013) |
| Cornusiine ou Eucalbanine A | *E. globulus* | Leaves | 0.23 | Boulekbache‐Makhlouf et al. (2013) |
| Eucalbanine | *E. globulus* | Leaves | 4.58 | Boulekbache‐Makhlouf et al. (2013) |
| Tellimagrandin I | *E. globulus* | Leaves | 1.43 | Boulekbache‐Makhlouf et al. (2013) |
| Tellimagrandin II | *E. globulus* | Leaves | 1.5 | Boulekbache‐Makhlouf et al. (2013) |
| Galloyl ester of methylellagic acid glucose | *E. globulus* | Leaves | 1.33 | Boulekbache‐Makhlouf et al. (2013) |
| Sideroxylonal | *E. globulus* | Leaves | 77.02 | Boulekbache‐Makhlouf et al. (2013) |
| Quercetin | *E. globulus* | Leaves | 0.00201 ± 0.00006 | Dezsi et al. (2015) |
| Quercetin-3-O-galactoside | *E. globulus* | Leaves | 0.66642 ± 0.0502 | Dezsi et al. (2015) |
| Isoquercitrin | *E. globulus* | Leaves | 0.03895 ± 0.00572 | Dezsi et al. (2015) |
| Rutin | *E. globulus* | Leaves | 0.04865 ± 0.00332 | Dezsi et al. (2015) |
| Myricetin | *E. globulus* | Leaves | 0.09234 ± 0.00021 | Dezsi et al. (2015) |
| Quercetin-3-O-rhamnoside | *E. globulus* | Leaves | 0.28783 ± 0.00212 | Dezsi et al. (2015) |
| Luteolin | *E. globulus* | Leaves | 0.0344 ± 0.00173 | Dezsi et al. (2015) |
| Apigenin | *E. globulus* | Leaves | 0.00285 ± 0.00002 | Dezsi et al. (2015) |
| Rutin | *E. globulus* | Leaves | 4.44 ± 0.33 | Almeida et al. (2009) |
| Quercetin-3-O-rhamnoside | *E. globulus* | Leaves | 2.41 ± 0.09 | Almeida et al. (2009) |
| Chlorogenic acid | *E. globulus* | Leaves | 4.49 ± 0.16 | Almeida et al. (2009) |
| Ellagic acid | *E. globulus* | Leaves | 2.68 ± 0.27 | Almeida et al. (2009) |
| Flavonol glycoside | *E. globulus* | Leaves | 9.85 ± 0.07 | Almeida et al. (2009) |
| Quinic acid | *E. globulus* | Bark | 1.46 | Santos et al. (2011) |
| Gallic acid | *E. globulus* | Bark | 3.41 | Santos et al. (2011) |
| Protocatechuic acid | *E. globulus* | Bark | 1.62 | Santos et al. (2011) |
| Methyl gallate | *E. globulus* | Bark | 0.68 | Santos et al. (2011) |
| Catechin | *E. globulus* | Bark | 6.57 | Santos et al. (2011) |
| Chlorogenic acid | *E. globulus* | Bark | 5.98 | Santos et al. (2011) |
| Ellagic acid | *E. globulus* | Bark | 4.95 | Santos et al. (2011) |
| Taxifolin | *E. globulus* | Bark | 1.48 | Santos et al. (2011) |
| Quercetin-hexoside | *E. globulus* | Bark | 0.15 | Santos et al. (2011) |
| Isorhamnetin | *E. globulus* | Bark | 3.98 | Santos et al. (2011) |
| Naringenin | *E. globulus* | Bark | 0.79 | Santos et al. (2011) |
| Bis(hexahydroxydiphenoyl)-glucose | *E. globulus* | Bark | 0.68 | Santos et al. (2011) |
| Galloyl-bis-HHDP-glucose | *E. globulus* | Bark | 7.23 | Santos et al. (2011) |
| Galloyl-hexahydroxydiphenoyl-glucose | *E. globulus* | Bark | 9.27 | Santos et al. (2011) |
| Digalloylglucose | *E. globulus* | Bark | 17.95 | Santos et al. (2011) |
| Isorhamnetin-hexoside | *E. globulus* | Bark | 1.53 | Santos et al. (2011) |
| Methylellagic acid-pentose | *E. globulus* | Bark | <LOD | Santos et al. (2011) |
| Myricetin-rhamnoside | *E. globulus* | Bark | 0.2 | Santos et al. (2011) |
| Aromadendrin-rhamnoside | *E. globulus* | Bark | <LOD | Santos et al. (2011) |
| Mearnsetin | *E. globulus* | Bark | 0.34 | Santos et al. (2011) |
| Phloridzin | *E. globulus* | Bark | <LOD | Santos et al. (2011) |
| Mearnsetin-hexoside | *E. globulus* | Bark | 1.07 | Santos et al. (2011) |
| Eriodictyol | *E. globulus* | Bark | 6.9 | Santos et al. (2011) |
| Luteolin | *E. globulus* | Bark | 2.31 | Santos et al. (2011) |
| Gallic acid | *E. camaldulensis* | Bark | 0.0332 | Abdelkhalek et al. (2020) |
| Chlorogenic acid | *E. camaldulensis* | Bark | 0.1017 | Abdelkhalek et al. (2020) |
| Ellagic acid | *E. camaldulensis* | Bark | 0.831 | Abdelkhalek et al. (2020) |
| Caffeic acid | *E. camaldulensis* | Bark | 0.1837 | Abdelkhalek et al. (2020) |
| p-coumaric acid | *E. camaldulensis* | Bark | 0.01 | Abdelkhalek et al. (2020) |
| Ferulic acid | *E. camaldulensis* | Bark | 0.0456 | Abdelkhalek et al. (2020) |
| Rutin | *E. camaldulensis* | Bark | 1.4678 | Abdelkhalek et al. (2020) |
| Myricetin | *E. camaldulensis* | Bark | 1.7542 | Abdelkhalek et al. (2020) |
| Quinol | *E. camaldulensis* | Bark | 3.0668 | Abdelkhalek et al. (2020) |
| p-Hydroxy benzoic acid | *E. camaldulensis* | Bark | 0.298 | Abdelkhalek et al. (2020) |
| Vanillic acid | *E. camaldulensis* | Bark | 0.4186 | Abdelkhalek et al. (2020) |
| Syringic acid | *E. camaldulensis* | Bark | 0.1308 | Abdelkhalek et al. (2020) |
| Vanillin | *E. camaldulensis* | Bark | 0.0241 | Abdelkhalek et al. (2020) |
| Benzoic acid | *E. camaldulensis* | Bark | 3.6382 | Abdelkhalek et al. (2020) |
| o-coumaric acid | *E. camaldulensis* | Bark | 0.0634 | Abdelkhalek et al. (2020) |
| Salicylic acid | *E. camaldulensis* | Bark | 1.603 | Abdelkhalek et al. (2020) |
| Cinnamic acid | *E. camaldulensis* | Bark | 0.0138 | Abdelkhalek et al. (2020) |
| Quinic acid | *E. marginata* | Leaves | 0.02518 ± 0.00004 | Djebbi et al. (2024) |
| Gallic acid | *E. marginata* | Leaves | 0.01722 ± 0.00005 | Djebbi et al. (2024) |
| Rutin | *E. marginata* | Leaves | 0.00213 ± 0.00006 | Djebbi et al. (2024) |
| Catechin | *E. marginata* | Leaves | 0.01342 ± 0.00008 | Djebbi et al. (2024) |
| Quercetin-3-O-rhamnoside | *E. marginata* | Leaves | 0.03106 ± 0.00003 | Djebbi et al. (2024) |
| Naringenin-7-O-neohesperidoside | *E. marginata* | Leaves | 0.02076 ± 0.00002 | Djebbi et al. (2024) |
| p-coumaric acid | *E. marginata* | Leaves | 0.00525 ± 0.00007 | Djebbi et al. (2024) |
| Quercetin-3-O-galactoside | *E. marginata* | Leaves | 0.04146 ± 0.00009 | Djebbi et al. (2024) |
| Luteolin-7-O-glucoside | *E. marginata* | Leaves | 0.0126 ± 0.00005 | Djebbi et al. (2024) |
| o-coumaric acid | *E. marginata* | Leaves | 0.00376 ± 0.00006 | Djebbi et al. (2024) |
| Rosmarinic acid | *E. marginata* | Leaves | 0.00426 ± 0.00007 | Djebbi et al. (2024) |
| Apigenin-7-O-glucoside | *E. marginata* | Leaves | 0.0046 ± 0.00001 | Djebbi et al. (2024) |
| Trans-cinnamic acid | *E. marginata* | Leaves | 0.28971 ± 0.00007 | Djebbi et al. (2024) |
| Quercetin | *E. marginata* | Leaves | 0.00084 ± 0.00005 | Djebbi et al. (2024) |
| Naringenin | *E. marginata* | Leaves | 0.00081 ± 0.00001 | Djebbi et al. (2024) |
| Acacetin | *E. marginata* | Leaves | 0.00019 ± 0.00003 | Djebbi et al. (2024) |
| p-coumaric acid | *E. marginata* | Leaves | 0.00467 ± 0.00006 | Hasni et al. (2021) |
| Quercetin-3-O-galactoside | *E. marginata* | Leaves | 0.09647 ± 0.00008 | Hasni et al. (2021) |
| Protocatechuic acid | *E. marginata* | Leaves | 0.0376 ± 0.00004 | Hasni et al. (2021) |
| Quinic acid | *E. marginata* | Leaves | 0.00165 ± 0.00002 | Hasni et al. (2021) |
| Gallic acid | *E. marginata* | Leaves | 0.02777 ± 0.00006 | Hasni et al. (2021) |
| Trans-ferulic acid | *E. marginata* | Leaves | 0.00012 ± 0.00003 | Hasni et al. (2021) |
| Quercetin-3-O-galactoside | *E. marginata* | Leaves | 0.09647 ± 0.00008 | Hasni et al. (2021) |
| Quercetin-3-O-rutinoside | *E. marginata* | Leaves | 0.00023 ± 0.00006 | Hasni et al. (2021) |
| Quercetin-3-O-rhamnoside | *E. marginata* | Leaves | 0.1811 ± 0.00005 | Hasni et al. (2021) |
| Naringenin-7-O-neohesperidoside | *E. marginata* | Leaves | 0.019 ± 0.00005 | Hasni et al. (2021) |
| Salviolinic acid | *E. marginata* | Leaves | 0.00075 ± 0.00005 | Hasni et al. (2021) |
| Trans-cinnamic acid | *E. marginata* | Leaves | 0.02199 ± 0.00001 | Hasni et al. (2021) |
| Quercetin | *E. marginata* | Leaves | 0.00321 ± 0.00001 | Hasni et al. (2021) |
| Naringenin | *E. marginata* | Leaves | 0.00123 ± 0.00009 | Hasni et al. (2021) |
| Gallic acid | *E. cinerea* | Bark | 0.176 | Grichi et al. (2025) |
| Catechin | *E. cinerea* | Bark | 0.231 | Grichi et al. (2025) |
| Quercetin-hexoside | *E. cinerea* | Bark | 0.151 | Grichi et al. (2025) |
| Caffeic acid | *E. cinerea* | Bark | 0.074 | Grichi et al. (2025) |
| Quercetin-3-O-rutinoside | *E. cinerea* | Bark | 0.757 | Grichi et al. (2025) |
| Myricetin-glucuronide | *E. cinerea* | Bark | 0.217 | Grichi et al. (2025) |
| Luteolin-diglucoside | *E. cinerea* | Bark | 0.231 | Grichi et al. (2025) |
| Galloylquinic acid | *E. cinerea* | Bark | 0.052 | Grichi et al. (2025) |
| Trigalloylquinic acid | *E. cinerea* | Bark | 0.260 | Grichi et al. (2025) |
| Gentisic glucoside acid | *E. cinerea* | Bark | 0.741 | Grichi et al. (2025) |
| Myricetin-diglucoside | *E. cinerea* | Bark | 0.453 | Grichi et al. (2025) |
| Quinic acid digalloy | *E. cinerea* | Bark | 0.155 | Grichi et al. (2025) |
| Myricetin-hexose | *E. cinerea* | Bark | 0.312 | Grichi et al. (2025) |
| Kaempherol-rutinoside | *E. cinerea* | Bark | 0.673 | Grichi et al. (2025) |
| Apigenin-diglucoside | *E. cinerea* | Bark | 0.984 | Grichi et al. (2025) |

* Romano et al. (2025) report phenolic compound contents as abundance values expressed as ×10³, based on the signal intensity detected by HPLC-QTOF-MS. <LOD: < Limit of Detection.
